# Comprehensive Phenotypic Characterization of Late Gadolinium Enhancement Predicts Sudden Cardiac Death in Coronary Artery Disease

**DOI:** 10.1016/j.jcmg.2022.10.020

**Published:** 2023-05

**Authors:** Richard E. Jones, Hassan A. Zaidi, Daniel J. Hammersley, Suzan Hatipoglu, Ruth Owen, Gabriel Balaban, Antonio de Marvao, François Simard, Amrit S. Lota, Ciara Mahon, Batool Almogheer, Lukas Mach, Francesca Musella, Xiuyu Chen, John Gregson, Laura Lazzari, Andrew Ravendren, Francisco Leyva, Shihua Zhao, Ali Vazir, Pablo Lamata, Brian P. Halliday, Dudley J. Pennell, Martin J. Bishop, Sanjay K. Prasad

**Affiliations:** a National Heart and Lung Institute, Imperial College London, United Kingdom; b Cardiovascular Magnetic Resonance Unit, Royal Brompton and Harefield Hospitals, Guy’s and St Thomas’ National Health Service Foundation Trust, London, United Kingdom; c Department of Biomedical Engineering, School of Biomedical & Imaging Sciences, King’s College London, United Kingdom; d Department of Medical Statistics, London School of Hygiene and Tropical Medicine, London, United Kingdom; e Department of Computational Physiology, Simula Research Laboratory, Oslo, Norway; f Department of Women and Children's Health, King's College London, London, United Kingdom; g British Heart Foundation Centre of Research Excellence, School of Cardiovascular Medicine and Sciences, King's College London, London, United Kingdom; h State Key Laboratory of Cardiovascular Disease, National Center for Cardiovascular Diseases, Chinese Academy of Medical Sciences and Peking Union Medical College, Beijing, China; i Aston Medical School, Aston University, Birmingham, United Kingdom

**Keywords:** computational analysis, coronary artery disease, late gadolinium enhancement cardiac magnetic resonance, sudden cardiac death

## Abstract

**Background:**

Late gadolinium enhancement (LGE) cardiac magnetic resonance (CMR) offers the potential to noninvasively characterize the phenotypic substrate for sudden cardiac death (SCD).

**Objectives:**

The authors assessed the utility of infarct characterization by CMR, including scar microstructure analysis, to predict SCD in patients with coronary artery disease (CAD).

**Methods:**

Patients with stable CAD were prospectively recruited into a CMR registry. LGE quantification of core infarction and the peri-infarct zone (PIZ) was performed alongside computational image analysis to extract morphologic and texture scar microstructure features. The primary outcome was SCD or aborted SCD.

**Results:**

Of 437 patients (mean age: 64 years; mean left ventricular ejection fraction [LVEF]: 47%) followed for a median of 6.3 years, 49 patients (11.2%) experienced the primary outcome. On multivariable analysis, PIZ mass and core infarct mass were independently associated with the primary outcome (per gram: HR: 1.07 [95% CI: 1.02-1.12]; *P =* 0.002 and HR: 1.03 [95% CI: 1.01-1.05]; *P =* 0.01, respectively), and the addition of both parameters improved discrimination of the model (Harrell’s C-statistic: 0.64-0.79). PIZ mass, however, did not provide incremental prognostic value over core infarct mass based on Harrell’s C-statistic or risk reclassification analysis. Severely reduced LVEF did not predict the primary endpoint after adjustment for scar mass. On scar microstructure analysis, the number of LGE islands in addition to scar transmurality, radiality, interface area, and entropy were all associated with the primary outcome after adjustment for severely reduced LVEF and New York Heart Association functional class of >1. No scar microstructure feature remained associated with the primary endpoint when PIZ mass and core infarct mass were added to the regression models.

**Conclusions:**

Comprehensive LGE characterization independently predicted SCD risk beyond conventional predictors used in implantable cardioverter-defibrillator (ICD) insertion guidelines. These results signify the potential for a more personalized approach to determining ICD candidacy in CAD.

Current tools to identify patients at high risk of sudden cardiac death (SCD) are limited. Specifically, left ventricular ejection fraction (LVEF) is an imprecise metric, and innovative approaches are required to identify arrhythmogenic substrate beyond this measure.^1^ SCD risk prediction is of notable importance for patients with coronary artery disease (CAD) because their underlying etiology alone confers an enhanced risk profile.^2^ It is therefore crucial to evaluate the utility of novel prediction tools to identify high-risk patients within this cohort.

In patients with chronic CAD, re-entrant ventricular tachycardia (VT) is the presumed mechanism underpinning the majority of SCD cases.^3^ Septa of replacement extracellular fibrosis (resultant from necrosing myocytes) perforating bundles of surviving myocytes can provide an arrhythmogenic milieu capable of facilitating a re-entry circuit.^4^ These areas of heterogeneous tissue, more recently termed the “peri-infarct” zone (PIZ) or “gray” zone, are typically located at the transition point between viable myocardium and compact scar and are hypothesized to contain the substrate for slow conduction and fixed/functional block that initiate and maintain VT.^3^ Implantable cardioverter-defibrillators (ICDs) can treat re-entrant arrhythmia and have been shown to protect against a high proportion of SCD.^5^ Decisions regarding primary prevention ICD insertion currently center around evaluation of New York Heart Association (NYHA) functional class alongside dichotomous assessment of left ventricular (LV) systolic function using an LVEF cutoff of 30% to 35%.^6^^,^^7^ Typically assessed at a solitary timepoint, this fails to take into account the dynamic nature of LVEF.^1^^,^^8^ The ever-increasing demand on clinical services represents a parallel issue, and a comprehensive assessment of SCD risk at a singular timepoint (remote from acute myocardial infarction) is desirable. Less dynamic features, which characterize the arrhythmogenic substrate, therefore offer promise.

Late gadolinium enhancement (LGE) cardiac magnetic resonance (CMR) noninvasively identifies dense myocardial fibrosis with high spatial resolution and has good histologic correlation in CAD models.^9^ Additionally, quantification of the PIZ by LGE-CMR has been shown to be associated with all-cause mortality^10^, ^11^, ^12^, ^13^ and ICD therapy,^14^ with more recent studies also evaluating the role of scar microstructure (eg, entropy) in ventricular arrhythmia.^15^ There remains, however, a paucity of data describing the application of complex scar analysis to predict SCD in prospectively recruited cohorts with a broad range of LVEF. Indeed, the utility of noninvasive imaging to predict SCD remains a key research need highlighted by both the American Heart Association and European Society of Cardiology.^6^^,^^7^ We performed LGE quantification, in combination with bespoke computational analysis of scar microstructure features, to provide a novel mechanistic interrogation into the drivers of SCD in prospectively investigated patients with CAD.

## Methods

### Study design

Patients referred to our center for evaluation of ischemic heart disease with LGE-CMR were recruited into a registry between August 2009 and January 2016. These patients were referred from local cardiology clinics in addition to a broad network of specialist and nonspecialist hospitals. The registry complied with the Declaration of Helsinki, and the National Research Ethics Service approved the protocol. All patients provided informed written consent. CMR was undertaken on a 1.5-T scanner (Sonata/Avanto, Siemens) using a standardized protocol on the day of recruitment or, in a minority of patients, at a prior date during disease work-up.^16^ The inclusion criteria for the study were: 1) severe epicardial CAD; 2) prior coronary revascularization; or 3) documented history of prior myocardial infarction (confirmed on CMR). Severe epicardial CAD was defined as ≥75% stenosis in the left main stem/proximal left anterior descending artery or ≥75% in 2 epicardial coronary arteries. Exclusion criteria were Class I indication for a secondary prevention ICD; myocardial infarction within 40 days before CMR; uninterpretable LGE imaging; severe primary valvular disease; previous valvular intervention; or a primary diagnosis of dilated cardiomyopathy, active myocarditis, cardiac sarcoidosis, hypertrophic cardiomyopathy, or infiltrative cardiomyopathy. Patients were followed up using health questionnaires alongside primary care and hospital documentation. All clinical outcomes were adjudicated by an independent panel of experienced cardiologists blinded to the LGE data. The a priori primary endpoint was a composite of SCD or aborted SCD. The full study design is detailed in the Supplemental Methods.

### CMR analysis

LGE quantification of the core infarct and adjacent PIZ was performed by a level 3 accredited CMR operator (S.H.) blinded to the clinical outcomes. Analysis was undertaken on CVI42 (Circle Cardiovascular Imaging Inc) using the full width at half maximum (FWHM) method. Core infarct was classified as any LV region with a signal intensity (SI) of ≥50% of the maximal SI found within the reference scar region of interest. The PIZ was defined as LV myocardium (adjacent to core infarct) with an SI between 35% and 50% of the maximally detected SI within the reference scar region of interest.

With harnessing of both the contoured LGE images and corresponding slice masks, morphologic and texture scar microstructure features were extracted using proprietary software as previously described by our group.^17^^,^^18^ The calculated scar microstructure features were: 1) transmurality; 2) radiality; 3) number of LGE components; 4) interface area; and 5) entropy (Table 1). A comprehensive description of the CMR analysis is provided in the Supplemental Methods.

**Table 1:** Scar Microstructure Features

Scar Feature	Feature Description
Transmurality^a^	The extent of spread of LGE emanating outward from the endocardium to epicardium, calculated using a ray tracing method
Radiality^a^	Quantification of the circumferential spread of LGE in relation to the center of the LV blood pool.
Number of components^a^	The number of distinct LGE components in a slice (eg, the number of islands of core or PIZ scar)
Interface area^a^	The extent (ie, area) of the border zone between myocardium and adjacent LGE
Entropy^b^	The level of disorder or heterogeneity within the LGE, calculated by applying standard Shannon entropy^15^

The 5 scar microstructure features that were extracted from the LGE images.

LGE = late gadolinium enhancement; LV = left ventricle; PIZ = peri-infarct zone.

a Morphologic feature.

b Texture feature.

### Statistical analysis

Baseline characteristics were summarized in the total cohort as frequency (%) for categorical variables and mean ± SD or median (IQR) where appropriate for continuous variables. Kaplan-Meier curves were plotted to describe the cumulative incidence of the primary outcome by tertiles of PIZ mass and core infarct mass over follow-up, compared using the log-rank test.

To investigate the utility of LGE quantification in the prediction of the primary outcome, we generated univariable and multivariable Cox regression models. The primary multivariable model was adjusted using binary cutoffs of an LVEF of <35% and NYHA functional class of >1 to align with current clinical guidelines for ICD insertion (model A).^7^ Competing risk analysis was performed using Fine-Gray subdistribution hazard modeling. Additionally, the incremental predictive value of PIZ mass was examined by calculating categorical (using thresholds of 0%-10%, 10%-20%, and 20%+ to stratify the level of risk) and category-free net reclassification indices. A secondary Cox regression model was also fitted (model B). To select the covariables in model B, a forward stepwise procedure was applied using a subset of variables in Table 2 with *P <* 0.10 as the criterion for inclusion, forcing in known predictors of the outcome (age, sex, and LVEF). To prevent the creation of an overly complex model, not all variables that were associated with the primary endpoint on univariable analysis were used in the multivariable model (eg, indexed LV mass and RVEF). In all models, core infarct mass and PIZ mass were subsequently added to assert whether either metric was independently associated with the primary outcome. Model performance was assessed using Harrell’s C-statistic. Forest plots were generated using core infarct mass and PIZ mass per 10 g to aid visual representation. Details of the statistical analysis for the secondary endpoints and sensitivity analyses are provided in the Supplemental Methods. All analysis was performed on Stata version 17 (StataCorp) and Python version 3.7.4 (Python Software Foundation).

**Table 2:** Baseline Characteristics

	All Patients	PIZ <8.8 g	PIZ ≥8.8 g	*P* Value
Demographics				
Age, y	64.4 ± 9.9	65.2 ± 9.5	63.6 ± 10.2	0.10
Female	61 (14.0)	41 (18.8)	20 (9.1)	0.004
White	357 (81.7)	178 (81.7)	179 (81.7)	0.98
BMI, kg/m^2^	27.8 ± 5.0	27.6 ± 4.7	28.0 ± 5.2	0.34
Heart rate, beats/min	69.3 ± 13.3	69.4 ± 14.1	69.3 ± 12.6	0.92
SBP, mm Hg	126.5 ± 19.3	130.8 ± 19.4	122.5 ± 18.3	<0.001
DBP, mm Hg	73.3 ± 11.7	75.3 ± 11.5	71.4 ± 11.7	<0.001
Significant CAD^a^	412 (95.4)	198 (91.2)	214 (99.5)	<0.001
CAD type				0.15
1 vessel	131 (31.8)	72 (36.4)	59 (27.6)	
2 vessels	117 (28.4)	51 (25.8)	66 (30.8)	
3 vessels	164 (39.8)	75 (37.9)	89 (41.6)	
History of MI	316 (72.3)	141 (64.7)	175 (79.9)	<0.001
Prior PCI	225 (51.5)	119 (54.6)	106 (48.4)	0.20
Prior CABG	121 (27.7)	58 (26.6)	63 (28.8)	0.61
Hypertension	231 (52.9)	116 (53.2)	115 (52.5)	0.88
Diabetes mellitus	128 (29.3)	59 (27.1)	69 (31.5)	0.31
Documented hypercholesterolemia	356 (81.5)	171 (78.4)	185 (84.5)	0.10
Documented family history of premature CAD	95 (21.7)	50 (22.9)	45 (20.5)	0.54
Smoking status				0.01
Yes	45 (10.3)	19 (8.8)	26 (11.9)	
Ex-smoker	242 (55.5)	109 (50.2)	133 (60.7)	
No	149 (34.2)	89 (41.0)	60 (27.4)	
Baseline AF	73 (16.7)	33 (15.1)	40 (18.3)	0.38
NYHA functional class				0.17
I	145 (33.3)	81 (37.3)	64 (29.4)	
II	197 (45.3)	95 (43.8)	102 (46.8)	
III or IV	93 (21.4)	41 (18.9)	52 (23.9)	
Medications				
Antithrombotic therapy	420 (96.1)	210 (96.3)	210 (95.9)	0.81
Beta-blocker	338 (77.3)	158 (72.5)	180 (82.2)	0.02
ACE inhibitor/ARB	368 (84.2)	168 (77.1)	200 (91.3)	<0.001
Lipid-lowering drug	386 (88.3)	188 (86.2)	198 (90.4)	0.17
CMR volumetric measurements				
LVEF, %	47.2 ± 16.8	55.2 ± 16.0	39.2 ± 13.3	<0.001
Indexed LV mass, g/m^2^	79.1 ± 24.6	72.1 ± 20.9	86.0 ± 26.0	<0.001
LVEDVI, mL/m^2^	106.5 ± 40.8	89.1 ± 28.9	123.7 ± 43.5	<0.001
RVEF, %	58.1 ± 12.7	59.4 ± 11.2	56.8 ± 13.9	0.03
CMR LGE characteristics				
Presence of infarct pattern LGE	378 (86.5)	159 (72.9)	219 (100.0)	<0.001
Predominant territory				0.010
Anterior	172 (45.5)	60 (37.7)	112 (51.1)	
Lateral	54 (14.3)	31 (19.5)	23 (10.5)	
Inferior	152 (40.2)	68 (42.8)	84 (38.4)	
PIZ mass, g	8.8 (4.2-14.4)	NA	NA	NA
Infarct core mass, g	17.6 (6.5-30.2)	6.4 (0.0-12.8)	29.0 (20.8-39.6)	<0.001

Values are mean ± SD, n (%), or median (IQR). Characteristics were compared between patients with a PIZ mass less than the median vs those with a PIZ mass of the median or greater using 2-sample Student’s *t*-test or Mann-Whitney *U* test for continuous variables and chi-square test or Fisher exact test for categorical variables.

ACE = angiotensin-converting enzyme; AF = atrial fibrillation; ARB = angiotensin II receptor blocker; BMI = body mass index; CABG = coronary artery bypass grafting; CAD = coronary artery disease; CMR = cardiac magnetic resonance; DBP = diastolic blood pressure; LVEDVI = indexed left ventricular end-diastolic volume; LVEF = left ventricular ejection fraction; MI = myocardial infarction; NA = not applicable; NYHA = New York Heart Association; RVEF = right ventricular ejection fraction; PCI = percutaneous coronary intervention; SBP = systolic blood pressure; other abbreviations as in Table 1.

a Patients with evidence of severe CAD or a history of prior coronary revascularization.

## Results

At baseline, 734 patients were assessed for eligibility, with the final cohort consisting of 437 patients (Supplemental Figure 1). The median interval between CMR and recruitment was 0 days (IQR: 0-0 days), the mean age was 64.4 ± 9.9 years, the mean LVEF was 47.2% ± 16.8%, and 412 (95%) patients had significant CAD or had previously undergone coronary revascularization. Patients were followed up for a median of 6.3 years (IQR: 5.0-7.9 years). PIZ mass and core mass were strongly correlated (Pearson’s correlation coefficient: *r* = 0.81). Baseline characteristics are described in Table 2 (with extended data in Supplemental Tables 1 and 2). Extended analysis is detailed in the Supplemental Results; reproducibility data for the scar quantification are shown in Supplemental Table 3.

### Primary outcome

#### Composite of SCD or aborted SCD: Core infarct mass and PIZ mass

**Figure 1:**
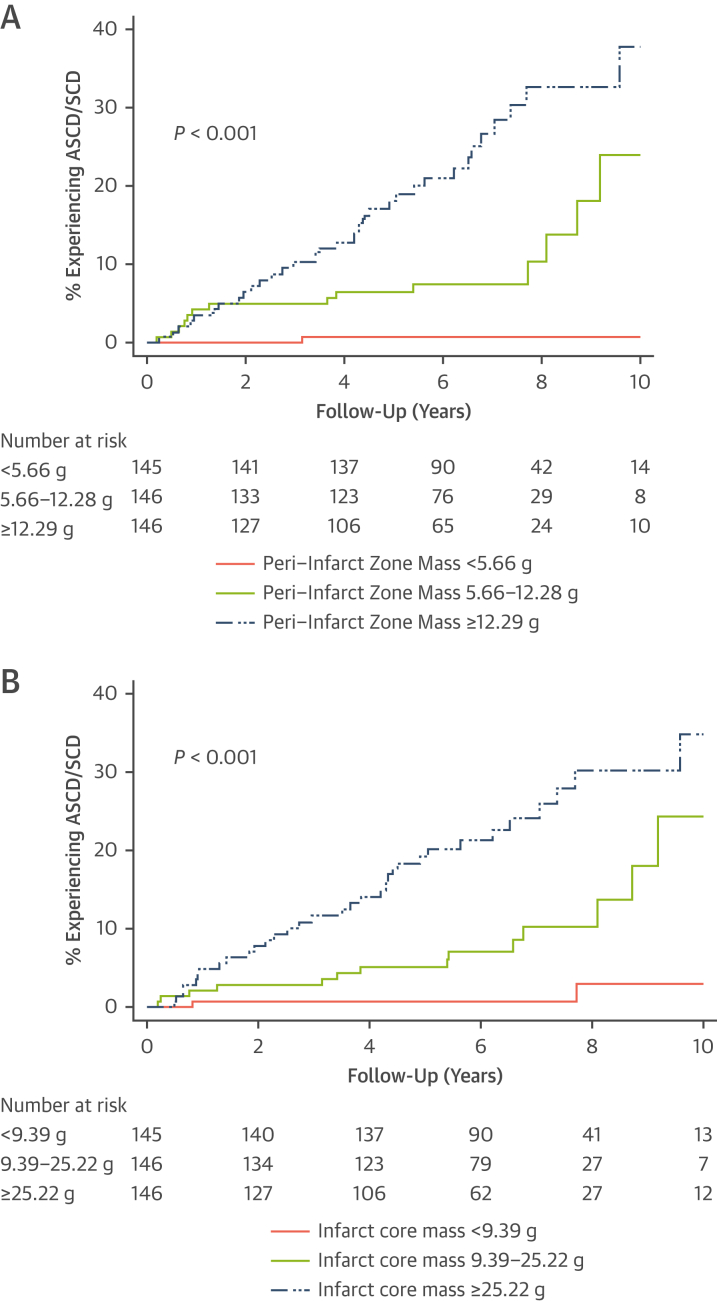
Kaplan-Meier Plots of the Primary Endpoint by Tertiles of Core Infarct Mass and PIZ Mass **(A)** Kaplan-Meier plots of the primary endpoint by tertiles of PIZ mass. **(B)** Kaplan-Meier plots of the primary endpoint by tertiles of core infarct mass. ASCD = aborted sudden cardiac death; PIZ = peri-infarct zone; SCD = sudden cardiac death.

At the 10-year follow-up, 49 patients (11.2%) had experienced the primary outcome (20 patients experiencing SCD and 29 patients experiencing aborted SCD). Autopsy results were obtained for 12 of the deaths assigned as SCD. In cases for which autopsy data were not available, SCD was diagnosed by the independent panel of cardiologists using standard endpoint definitions.^19^ Cumulative incidence of the primary outcome by tertiles of PIZ mass suggest that patients in higher tertiles had an increased risk of the primary outcome (10-year risk: 0.7%, 24.0%, and 37.8% for patients with a PIZ mass of <5.66 g, 5.66-12.28 g, and ≥12.29 g, respectively; *P <* 0.001) (Figure 1). Similarly, patients in the higher tertiles of core mass had an increased risk of the primary outcome (10-year risk: 3.7%, 24.0%, and 34.6% for patients with a core mass of <9.39 g, 9.39-25.21 g and ≥25.22 g, respectively; *P <* 0.001) (Figure 1). On univariable analysis, an increase in PIZ mass and core infarct mass was significantly associated with an increased risk of the primary outcome (per gram: HR: 1.12 [95% CI: 1.09-1.15]; *P <* 0.001 and HR: 1.05 [95% CI: 1.04-1.06]; *P <* 0.001, respectively). Additionally, a decrease in LVEF was significantly associated with an increased risk of the primary outcome on univariable analysis (per %: HR: 0.96 [95% CI: 0.94-0.97]; *P <* 0.001).

**Figure 2:**
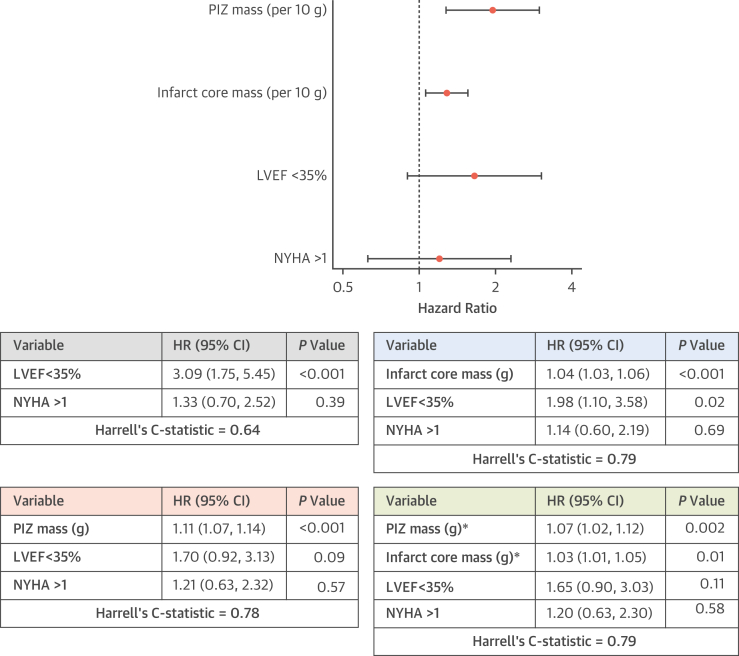
Multivariable Cox Regression Analysis (Model A) for the Primary Endpoint With Subsequent Addition of the LGE Quantification Data **(Top)** Forest plot of the final multivariable Cox model using an LVEF of <35%, NYHA functional class of >1, core infarct mass, and PIZ mass. Data are presented using LGE results per 10 g. **(Middle and bottom)** Multivariable Cox models using binary cutoffs of an LVEF of <35% and NYHA functional class of >1 with subsequent addition of the LGE quantification data. **∗**Results per 10 g: for PIZ mass, HR: 1.95 [95% CI: 1.28-2.98]; *P =* 0.002 and for core infarct mass, HR: 1.28 [95% CI: 1.06-1.56]; *P =* 0.01. LGE = late gadolinium enhancement; LVEF = left ventricular ejection fraction; NYHA = New York Heart Association; other abbreviation as in Figure 1.

After adjustment for the variables in model A (LVEF of <35% and NYHA functional class of >1), both PIZ mass and core infarct mass remained independently associated with the primary outcome (per gram: HR: 1.07 [95% CI: 1.02-1.12]; *P =* 0.002 and HR: 1.03 [95% CI: 1.01-1.05]; *P =* 0.01, respectively) (Figure 2) and improved the discrimination ability of the model (C-statistic: 0.64-0.79). Model performance was similar with either the addition of core infarct mass or PIZ mass alone or the simultaneous addition of both scar metrics (Figure 2). Additionally, there was no overall net reclassification improvement after adding PIZ mass to the risk model of LVEF of <35%, NYHA of >1, and core infarct mass (categorical net reclassification index: 0.01; 95% CI: −0.52 to 0.56) (Supplemental Figure 2). Severely impaired LVEF was not associated with the primary endpoint on multivariable analysis (HR: 1.65 [95% CI: 0.90-3.03]; *P =* 0.11) (Figure 2). Indeed, removing LVEF from the final model resulted in no change in the C-statistic (0.79-0.79). Additionally, PIZ mass and core infarct mass remained independently associated with the primary endpoint after accounting for the competing risk of nonsudden death (per gram: subdistribution HR: 1.07 [95% CI: 1.03-1.12]; *P =* 0.001 and subdistribution HR: 1.03 [95% CI: 1.01-1.04]; *P =* 0.003, respectively) (Supplemental Figure 3). On sensitivity analyses, the estimated HRs were similar after adjustment for LVEF of <35%, NYHA functional class of >1: and 1) relative PIZ mass and core infarct mass expressed as a percentage of LV mass (per %: HR: 1.11 [95% CI: 1.06-1.17]; *P <* 0.001 and HR: 1.05 [95% CI: 1.02-1.08]; *P <* 0.001, respectively); or 2) PIZ mass and core infarct mass when only the 378 (87%) patients with infarct pattern LGE were included (per gram: HR: 1.06 [95% CI: 1.02-1.11]; *P =* 0.005 and HR: 1.02 [95% CI: 1.00-1.04]; *P =* 0.02, respectively).

Using model B, PIZ mass and core infarct mass remained independently associated with the primary outcome after adjustment for baseline covariates (per gram: HR: 1.07 [95% CI: 1.02-1.12]; *P =* 0.005 and HR: 1.02 [95% CI: 1.00-1.05]; *P =* 0.03, respectively) (Supplemental Figure 4) and improved the discrimination ability of the model to predict the primary outcome (C-statistic: 0.76-0.82). LVEF was not associated with the primary endpoint on multivariable analysis (per %: HR: 0.99 [95% CI: 0.97-1.02]; *P =* 0.63) (Supplemental Figure 4). Model performance was similar with either the addition of core infarct mass or PIZ mass alone or the simultaneous addition of both scar metrics (Supplemental Figure 4). A summary of univariable and multivariable Cox regression analyses is detailed in Supplemental Table 4.

#### Composite of SCD or aborted SCD: Scar microstructure analysis

**Figure 3:**
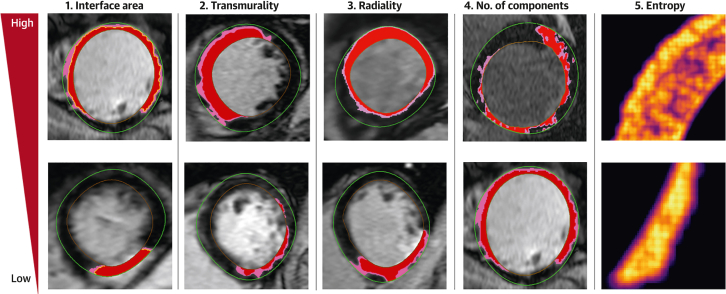
Scar Microstructure Features Extracted Using Computational Analysis of the LGE Images Example LGE-CMR images demonstrating the 5 scar microstructure feature groups. **(Top row)** High feature value. **(Bottom row)** Low feature value. Core scar is demonstrated in **red** and the PIZ in **pink** in **images 1 to 4**. The core infarct interface area is represented by the **yellow line** in **image 1**. CMR = cardiac magnetic resonance; other abbreviations as in Figures 1 and 2.

In examining the scar microstructure, each of the 5 features in Table 1 was separately calculated for: 1) core infarct; 2) PIZ; and 3) total scar (core infarct + PIZ). Figure 3 shows example images highlighting LGE slices with high and low feature values.

**Figure 4:**
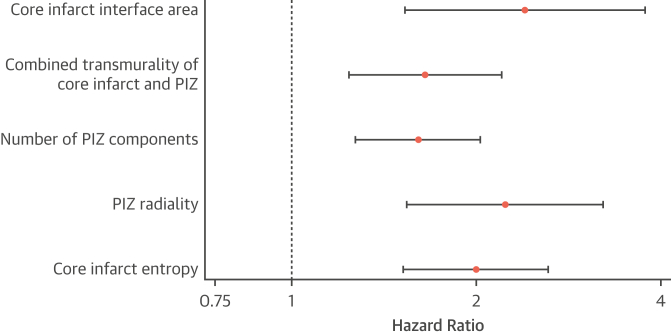
Multivariable Analysis of the Scar Microstructure Features for the Primary Outcome Individual Cox regression models for the scar microstructure features with the largest effect estimates within each group. Each model is adjusted for an LVEF of <35% and NYHA functional class of >1. Abbreviations as in Figures 1 and 2.

All scar microstructure features were associated with the primary endpoint on univariable and multivariable Cox regression analysis, the latter after adjustment for LVEF of <35% and NYHA functional class of >1 (Supplemental Table 5). Figure 4 shows the scar microstructure features with the largest effect estimates. After the addition of core infarct mass and PIZ mass to the Cox regression models, no scar microstructure feature remained significantly associated with the primary endpoint (Supplemental Table 6).

### Secondary outcomes

#### Major heart failure event

During follow-up, 78 (17.9%) patients experienced a major heart failure event. On multivariable analysis, there was no significant association between either scar metric and this secondary endpoint (PIZ mass per gram: HR: 1.02 [95% CI: 0.98-1.06]; *P =* 0.35; core infarct mass per gram: HR: 1.00 [95% CI: 0.98-1.02]; *P =* 0.77) (Supplemental Figure 5).

#### All-cause mortality

There were 138 (31.6%) deaths during the follow-up period (92 cardiovascular deaths, 46 noncardiovascular deaths). On multivariable analysis, PIZ mass was independently associated with mortality (per gram: HR: 1.04 [95% CI: 1.01-1.08]; *P =* 0.02) (Supplemental Figure 6). Core infarct mass was not significantly associated with the endpoint when both scar metrics were included in the model (per gram: HR: 1.00 [95% CI: 0.98-1.02]; *P =* 0.96) (Supplemental Figure 6).

#### Composite of sudden cardiac death or aborted sudden cardiac death in patients with an LVEF of ≥35%

Of 319 patients with an LVEF of ≥35%, 25 experienced SCD or aborted SCD. The cumulative incidences of the outcome by tertiles of both PIZ mass and core infarct mass suggest that patients in higher tertiles had an increased risk of the primary outcome (Supplemental Figure 7). On univariable analysis, both PIZ mass and core infarct mass were associated with the endpoint (per gram: HR: 1.09 [95% CI: 1.04-1.14]; *P <* 0.001 and HR: 1.04 [95% CI: 1.02-1.06]; *P <* 0.001, respectively). After adjustment for age, sex, and LVEF, neither PIZ mass nor core infarct mass remained significantly associated with the primary endpoint when both were included in the multivariable model (per gram: HR: 1.02 [95% CI: 0.94-1.11]; *P =* 0.58 and HR: 1.03 [95% CI: 0.99-1.07]; *P =* 0.13, respectively) (Supplemental Figure 7).

Scar microstructure analysis in patients with an LVEF of ≥35% highlighted 3 features that were associated with the primary endpoint on multivariable analysis (adjusted for age, sex, and LVEF): transmurality, interface area, and entropy (Supplemental Table 7). After the addition of core infarct mass and PIZ mass to the Cox regression models, 1 specific scar microstructure feature remained associated with the primary endpoint: combined transmurality of core infarct and the PIZ (HR: 2.29 [95% CI: 1.04-5.01]; *P =* 0.04). Additional results are detailed in Supplemental Tables 8 and 9.

## Discussion

To our knowledge, this is the first study assessing the utility of multiscale myocardial scar characterization by CMR to predict SCD in prospectively investigated patients with CAD. The cohort represents a real-world data set of typical patients with chronic coronary artery disease and a full spectrum of LV systolic function. The principal findings from our study are as follows (Central Illustration):

• PIZ mass and core infarct mass independently predict SCD after adjustment for clinical parameters used in ICD insertion decisions; importantly, however, PIZ mass does not have incremental prognostic value over core infarct mass.
• Reduced LVEF does not predict SCD when LGE quantification data are included in the multivariable models.
• Neither PIZ mass nor core infarct mass was associated with major heart failure events on multivariable analysis.
• Computational analysis identified a group of clinically plausible scar microstructure features that are associated with SCD; however, these metrics did not independently predict SCD after adjustment for total scar mass.

**Central Illustration:**
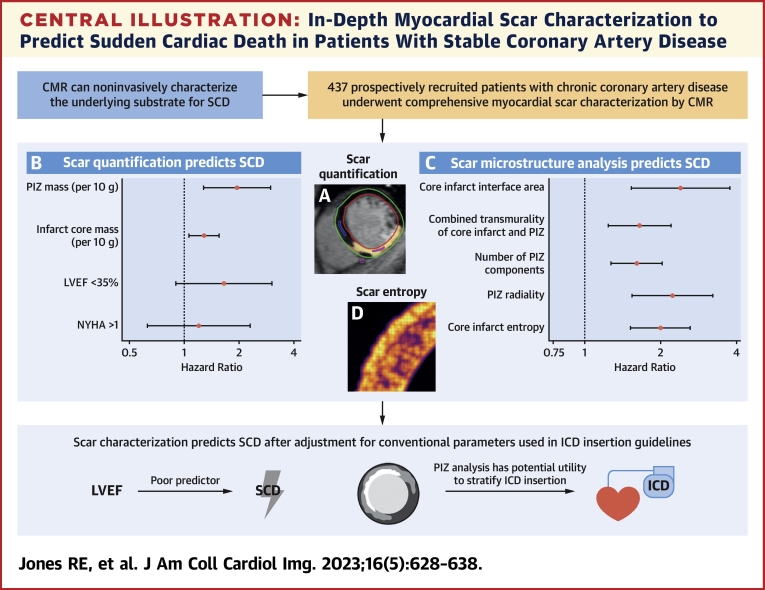
In-Depth Myocardial Scar Characterization to Predict Sudden Cardiac Death in Patients With Stable Coronary Artery Disease **(A)** Core infarct mass and PIZ mass independently predicted SCD after adjustment for parameters used in ICD insertion decisions. **(B)** PIZ mass and core infarct mass are presented per 10 g. **(C)** Computational LGE analysis extracted a set of morphologic and texture scar features that were associated with SCD on multivariable analysis (each model adjusted for LVEF of <35% and NYHA functional class of >1). **D** illustrates the texture-related feature, scar entropy. CMR = cardiac magnetic resonance; ICD = implantable cardioverter-defibrillator; LGE = late gadolinium enhancement; LVEF = left ventricular ejection fraction; NYHA = New York Heart Association; PIZ = peri-infarct zone; SCD = sudden cardiac death.

### LGE-CMR predictors of SCD in stable CAD

Multiple observational studies in CAD cohorts have demonstrated the role of PIZ quantification by LGE-CMR to identify patients at increased risk of all-cause mortality,^10^, ^11^, ^12^, ^13^ inducibility of VT during electrophysiology study,^20^ and appropriate ICD therapy.^14^^,^^21^, ^22^, ^23^ The majority of these studies either restricted recruitment to patients with impaired LVEF or those with prior ICD insertion. Additionally, in the studies including individuals with an existing secondary prevention ICD indication, the novel CMR metrics were unlikely to alter management decisions. Zegard et al^24^ recently published a retrospective study assessing the association between LGE and SCD in a cohort of CAD patients with a broad range of LVEF. In our study, using a prospectively recruited cohort, we build on the signal that they described in their retrospective registry, demonstrating that both core infarct mass and PIZ mass independently predict SCD. Importantly, however, PIZ mass does not improve risk prediction beyond core infarct mass based on Harrell’s C-statistic or risk reclassification analysis; the data suggest a lack of routine role for PIZ mass quantification in addition to infarct mass quantification for the determination of ICD candidacy.

Reduction in LVEF was not associated with the primary endpoint following the addition of the core infarct mass and PIZ mass to the multivariable models. This aligns with prior data in CAD cohorts detailing the limitation of LVEF to predict clinical outcomes after adjustment for LGE.^25^ These results are clinically relevant because LVEF calculation remains the central measure used in ICD insertion decisions,^6^^,^^7^ driven by inclusion criteria of the seminal trials assessing the utility of primary prevention ICD therapy.^26^^,^^27^ It is well appreciated, however, that impaired LVEF does not directly identify arrhythmogenic myocardial substrate. Up to 70% of SCD cases in CAD populations occur in subjects without severely reduced LVEF,^28^ and there remains a paucity of evidence identifying a convincing causal relationship between LVEF and SCD.^29^ Conversely, myocardial fibrosis is a mechanistically plausible metric in SCD prediction and may also represent a relatively static parameter in patients who do not experience a subsequent myocardial infarction.

Finally, neither core infarct mass nor PIZ mass predicted major heart failure events on multivariable analysis. Additionally, there was a significant association between both scar metrics and the primary endpoint after the competing risk of nonsudden death was accounted for. These results highlight the potential utility of LGE quantification as a precision tool in event prediction, hypothetically targeting ICD insertion to CAD patients with high future arrhythmic risk and lower risk of death from nonsudden causes. Importantly, the discrimination abilities of the multivariable models were similar following the addition of core infarct mass or PIZ mass alone or the simultaneous addition of both scar metrics, potentially suggesting that quantification of only 1 scar parameter may be needed to improve ICD candidacy.

### Scar microstructure analysis

Computational analysis of the LGE images permitted mechanistic interrogation of the key morphologic and texture-related scar features that predicted SCD, providing an additional layer of granularity above the raw quantification data.

Specifically, 2 of the morphologic features have a growing evidence base describing their association with ventricular arrhythmia. First, the interface area describes the extent of the border between LGE and adjacent tissue. This parameter has been found to be associated with major arrhythmic events in dilated cardiomyopathy, with simulation modeling describing a plausible mechanism of action to promote unidirectional conduction block.^18^ We show, for the first time to our knowledge, a significant association between scar interface area and SCD in patients with CAD. Second, critical VT isthmus sites have been found in close proximity to areas of increasingly transmural scar.^30^ Our study demonstrates the novel utility of scar transmurality to predict SCD in patients with CAD.

Recent studies have also demonstrated the association between the texture-related feature, scar entropy, and ventricular arrhythmia.^15^ Scar entropy describes the level of disorder or heterogeneity within a region of myocardial fibrosis.^15^ Our study is the first to demonstrate the utility of this microstructure feature to predict SCD in prospectively investigated patients with a broad range of LVEF.

Inclusion of both core infarct mass and PIZ mass in the multivariable models resulted in no scar microstructure feature remaining independently associated with the primary outcome. This may be explained by individual microstructure features scaling together, resulting in a combined signal that is adequately captured by raw quantification of core infarct and the PIZ, potentially suggesting a limited role for the extraction of morphologic and texture-related scar features in clinical practice. Importantly, however, in patients with an LVEF of ≥35%, high combined transmurality of the core infarct and PIZ identified patients at increased risk of SCD after inclusion of the LGE quantification data to the Cox regression model. Further mechanistic studies are required to fully dissect the role of this microstructure feature in arrhythmogenesis.

### Study limitations

This is a single-center study, and thus, the generalizability of the results may be questioned. However, our results align with previous prospective studies assessing all-cause mortality,^10^ and the patient cohort represents a real-world data set, referred from local cardiology clinics and a broad base of secondary and tertiary hospitals. Our study contains a high percentage of male and White patients, and thus, the results may not be applicable to female patients and non-White populations. We did not assess myocardial ischemia; however, the a priori hypothesis was to evaluate the utility of LGE characterization to predict SCD in stable CAD patients. Reflecting this, our cohort predominantly contained patients with mild ischemic symptoms (64% had Canadian Cardiovascular Society angina class 1, and 26% of patients had Canadian Cardiovascular Society class 2).

Concerns regarding the ability of 2-dimensional LGE to accurately characterize the PIZ have been raised. This principally surrounds the issue of partial voluming where imaged voxels contain both cleanly demarcated core scar and adjacent normal myocardium. This results in an intermediate signal because of the limited spatial resolution and not because of a region of viable myocytes interspersed with collagen. Future studies harnessing 3-dimensional LGE would allow for whole-heart coverage with reduced voxel dimensions and improved spatial resolution. The elimination of slice gaps would permit more detailed characterization of the relationship of LGE features along the slice direction (eg, increasing the confidence to describe potential VT conduction channels).

LGE quantification was only performed using the FWHM method, and recent retrospective studies have highlighted the association between LGE quantification by SD approaches and SCD.^24^ The FWHM approach, however, has the highest degree of reproducibility compared to other methods for quantifying LGE,^31^ and the majority of PIZ outcome studies have incorporated this methodology.^10^ Additionally, we only performed LGE quantification on a single platform, and therefore, the generalizability to other vendors is potentially limited.

## Conclusions

Our study provides novel prospective data demonstrating the value of myocardial fibrosis characterization by CMR to predict SCD in a cohort of stable CAD patients. We also highlight the limitation of LVEF calculation in SCD risk prediction. Multicenter trials should now be considered using appropriate cutoffs for these LGE metrics, above which patients are randomized to ICD insertion or conventional medical therapy.

Perspectives **COMPETENCY IN PATIENT CARE AND PROCEDURAL SKILLS:** Comprehensive LGE characterization can predict SCD in prospectively recruited patients with stable CAD and a broad range of LVEF. Additionally, numerous shape-based morphologic and texture scar microstructure features are associated with SCD, providing a degree of mechanistic interrogation beyond standard LGE characterization.

**TRANSLATIONAL OUTLOOK:** In-depth LGE characterization offers the potential to replace severe LV systolic impairment as the central metric used to identify primary prevention ICD candidates in patients with stable CAD.

## Funding Support and Author Disclosures

This work was supported by a National Heart and Lung Institute Foundation grant awarded to Drs Prasad and Jones. This work was also supported by a British Heart Foundation grant (FS/ICRF/21/26019) awarded to Dr Halliday and an Engineering and Physical Sciences Research Council grant (2018/19 DTP - EP/R513064/1) awarded to Mr Zaidi. Dr Leyva is a consultant with and has received research funding from Medtronic Inc, Boston Scientific, Abbott, Microport, and Biotronik. All other authors have reported that they have no relationships relevant to the contents of this paper to disclose.
