# Supplementary material for: Comprehensive Phenotypic Characterization of Late Gadolinium Enhancement Predicts Sudden Cardiac Death in Coronary Artery Disease
Source: JACC Cardiovasc Imaging. 2023 May;16(5):628–38. doi: 10.1016/j.jcmg.2022.10.020 (PMC10151254; doi:10.1016/j.jcmg.2022.10.020)
Supplement: Supplemental Data [file mmc1.docx]

## **Supplemental Methods**

### Additional inclusion/exclusion information

The 40-day post MI exclusion window was chosen in view of the impact of acute myocyte necrosis on LGE and international guidelines mandating this period of convalescence before consideration of primary prevention ICD therapy. No patients had an ICD implanted prior to CMR.

### Extended LGE-CMR protocol

The LGE datasets were acquired following intravenous injection of gadopentetate dimeglumine or gadobutrol (0.1mmol/Kg). An inversion recovery gradient echo sequence was subsequently undertaken at 10mins, typically in two phase encoding directions with parameters as described previously (1). The slice thickness was 8mm with a 2mm gap, resulting in an in-plane resolution of ~2.2mm x ~1.6mm. Inversion times were optimised to ensure adequate nulling of normal myocardium (characteristically between 280ms and 400ms).

### Extended description of the CMR analysis for LGE quantification.

Magnitude reconstructions of the gradient echo sequences were analysed. Initial quality control of each LGE study was undertaken and patients with suboptimal scans were excluded. Epicardial and endocardial LV contours were drawn from the short-axis LGE slices and a region of interest (ROI) was created within an area of infarct pattern hyperenhancement. Core infarct was then classified as any LV region with a signal intensity (SI) of ≥50% of the maximal SI found within the reference ROI. The PIZ was defined as LV myocardium (adjacent to core infarct) with SI between 35%-50% of the maximally detected SI within the reference ROI. Exclusion zones were applied to disregard regions that were erroneously identified as LGE. In the slices where no overt LGE was present, core mass and PIZ mass were imputed as 0g.

### Extended CMR analysis of the scar microstructure

The LGE slices were reviewed (RJ, HS) and contours optimised for computational analysis where necessary (e.g. to ensure the contours were not interrupted at the LV outflow tract). Harnessing both the contoured LGE images and corresponding slice masks, morphological and texture scar microstructure features were extracted using similar methodology on proprietary software as previously described by our group (2,3). The calculated scar microstructure features were i) transmurality; ii) radiality; iii) number of LGE components; iv) interface area; v) entropy (see Table 1 for list of feature descriptions). Specifically, interface area describes the extent of the interface/border between either core infarct or the PIZ and adjacent tissue or total scar (core infarct + PIZ) and adjacent myocardium, in both cases excluding the bounding epicardial/endocardial surfaces. Entropy represents the degree of complexity within an area of interest. In LGE analysis, entropy quantifies the heterogeneity in SI across a scar region; a uniform area of scar would have minimal difference in SI across the region, with a relatively low entropy value. A heterogenous region of scar would have a wider SI range, resulting in a higher entropy value.

Each scar microstructure feature was computed separately for: a) core infarct; b) PIZ; c) total scar (i.e. core infarct + PIZ). The individual slice results were then aggregated across the short-axis stack to better represent LGE topology throughout the LV, where applicable. All scripts for the microstructure analysis can be made available on reasonable request to the corresponding author.

### Extended follow up protocol and endpoint definitions

Clinical data was requested from general practitioners and the relevant hospitals. ICD reports, death certificates and post-mortem results were requested as appropriate. Survival status was confirmed via the UK NHS Digital service to ensure that no deaths were omitted. The duration of follow-up was determined from the date of CMR until an endpoint was confirmed or until the most recent patient contact date. Event times were calculated from the date of the preceding CMR up until a maximum of 10 years. SCD was defined as a death that occurred unexpectedly, including scenarios where symptom duration was ≤1hr, following an identified arrhythmia/unsuccessful resuscitation or in circumstances where the patient was witnessed alive ≤24hr prior to death and without another identifiable cause of death (4). Aborted SCD was defined as appropriate ICD shock for a ventricular tachyarrhythmia, effective resuscitation following ventricular fibrillation or haemodynamically unstable VT requiring electrical cardioversion (5). The prespecified secondary endpoints included: i) major heart failure (HF) composite of HF hospitalisation (admission to hospital of ≥24hr/encompassing 1 calendar day requiring initiation or escalation of HF therapies), HF death (in the context of progressive clinical features of HF), or cardiac transplantation/left ventricular assist device insertion, ii) all-cause mortality and iii) SCD or aborted SCD in patients with a LVEF ≥35%.

### Extended detail regarding the statistical analysis.

Reclassification of patient risk for the primary outcome was performed using net reclassification indices. Predicted overall risk of SCD/aborted SCD was first determined on the basis of a model using LVEF <35% and NYHA >1. The relative improvement in patient reclassification associated with PIZ mass was then assessed. Subsequently, predicted overall risk of SCD/aborted SCD was determined on the basis of a model using LVEF <35%, NYHA >1 and core infarct mass. Again, the relative improvement in patient reclassification associated with PIZ mass then assessed. In both analyses, reclassification was examined using thresholds of 0-10%, 10-20% and 20%+ to stratify level of risk (categorical NRI) and by calculating the category-free net reclassification index (continuous NRI).

To select the covariables used in Model B, a forward stepwise procedure was applied using a subset of variables in Table 2 with p<0.10 as the criterion for inclusion, forcing in known predictors of the outcome from existing literature (age, sex and LVEF). A limited number of robust variables were chosen to prevent creating an overly complex model. Not all variables that were associated with the primary endpoint on univariable analysis were used in the multivariable model (e.g., indexed LV mass and RVEF). In Model B, LVEF was measured as a continuous variable. The rest of the analysis plan for the primary endpoint is detailed in the main text.

The secondary endpoints were assessed using multivariable Cox regression modelling, selecting variables using a forward stepwise approach (as described in Model B). Subgroup analysis of patients with LVEF ≥35% was solely adjusted for age, sex and LVEF. The scar results are presented in grams reflecting the utility of absolute scar mass to predict SCD highlighted in a large, but retrospective, CMR study.(6) In addition, sensitivity analyses were conducted to: i) Assess the association between relative scar mass (expressed as a percentage of LV mass) and the primary outcome; ii) Evaluate the impact of excluding patients without infarct pattern LGE on the primary outcome; iii) Evaluate the association between total fibrosis mass (sum of core infarct mass and PIZ mass) and the primary endpoint; iv) Assess the association between scar mass and the primary endpoint after adjusting for future MI or subsequent coronary revascularisation as time-varying covariates.

To assess the association between the scar microstructure features and the primary outcome we generated univariable and multivariable Cox regression models, the latter adjusted for LVEF <35% and NYHA class >1. Core infarct mass and PIZ mass were then added to each model to assess whether any microstructure feature was independently associated with the primary outcome following inclusion of the traditional LGE quantification results.

## **Supplemental Results**

Overall, 412 (95%) patients had significant CAD or had previously undergone coronary revascularisation. All 25 (5%) patients without documented significant CAD/previous coronary revascularisation had a history of prior myocardial infarction confirmed on CMR.

Additional analyses were undertaken to assess the positive predictive value (PPV) and negative predictive value (NPV) of PIZ mass for subsequent SCD/aborted SCD, stratifying by LVEF. As the predictive values depend on the prevalence of the endpoint in the study population, and because the prevalence of our primary endpoint is relatively low (11%), the NPV will be higher and the PPV will be lower (Supplemental Table 8). We additionally assessed the PPV of LVEF <35% (the central metric used in international guidelines to determine primary prevention ICD candidacy) for predicting the primary endpoint. The results importantly demonstrate the increased performance of PIZ mass as compared to severely reduced LVEF in predicting SCD/aborted SCD (Supplemental Table 8).

Overall, 412 (94%) patients underwent CMR as an ambulatory outpatient visit, with the assessment of cardiac function and scar burden as the primary indication in 364 (83%) patients (Supplemental Table 9). During the study, 113 patients underwent ICD implantation (102 patients for primary prevention and 11 patients who developed a secondary indication during follow-up).

In addition to the sensitivity analyses for the primary endpoint detailed in the manuscript, the estimated HRs were also similar after adjusting for LVEF <35%, NYHA>1 and: i) PIZ mass and core infarct mass after adjusting for future revascularisation (n=161) as a time-updated covariate (per gram: HR 1.07, 95% CI 1.02-1.11, P=0.002 and HR 1.03, 95% CI 1.01-1.05, P=0.009 respectively); ii) PIZ mass and core infarct mass after adjusting for subsequent MI (n=32) as a time-varying covariate (per gram: HR 1.07, 95% CI 1.03-1.12, P=0.002 and HR 1.03, 95% CI 1.01-1.05, P=0.009 respectively); iii) total fibrosis mass as a sum of core infarct mass and the PIZ mass (per gram: HR 1.04, 95% CI 1.03-1.05, P<0.001).

| \| Supplemental Table 1: Baseline characteristics, stratified by the primary outcome. \| \| \| \| \| \| --- \| --- \| --- \| --- \| --- \| \| **Variable** \| **All patients** \| **No SCD/aSCD** \| **SCD/aSCD** \| **P-value** \| \| **Demographics** \|  \|  \|  \|  \| \| Age (years) \| 64.4 (9.9) \| 64.7 (10.0) \| 62.0 (8.5) \| 0.07 \| \| Female \| 61 (14.0) \| 59 (15.2) \| 2 (4.1) \| 0.03 \| \| Caucasian \| 357 (81.7) \| 178 (81.7) \| 179 (81.7) \| 0.98 \| \| BMI (kg/m2) \| 27.8 (5.0) \| 27.6 (4.9) \| 29.1 (5.2) \| 0.05 \| \| Heart rate (bpm) \| 69 (13) \| 69 (13) \| 71 (15) \| 0.52 \| \| SBP (mmHg) \| 127 (19) \| 127 (19) \| 123 (21) \| 0.20 \| \| DBP (mmHg) \| 73 (12) \| 74 (12) \| 72 (12) \| 0.50 \| \| Significant CAD* \| 412 (95.4) \| 365 (95.1) \| 47 (97.9) \| 0.37 \| \| CAD type \|  \|  \|  \| 0.15 \| \| *Single vessel* \| 131 (31.8) \| 118 (32.3) \| 13 (27.7) \|  \| \| *2 vessels* \| 117 (28.4) \| 102 (27.9) \| 15 (31.9) \|  \| \| *3 vessels* \| 164 (39.8) \| 145 (39.7) \| 19 (40.4) \|  \| \| History of MI \| 316 (72.3) \| 275 (70.9) \| 41 (83.7) \| 0.06 \| \| Prior PCI \| 225 (51.5) \| 205 (52.8) \| 20 (40.8) \| 0.11 \| \| Prior CABG \| 121 (27.7) \| 103 (26.5) \| 18 (36.7) \| 0.13 \| \| Hypertension \| 231 (52.9) \| 205 (52.8) \| 26 (53.1) \| 0.98 \| \| Diabetes mellitus \| 128 (29.3) \| 111 (28.6) \| 17 (34.7) \| 0.38 \| \| Documented hypercholesterolemia \| 356 (81.5) \| 314 (80.9) \| 42 (85.7) \| 0.42 \| \| Documented family history of premature CAD \| 95 (21.7) \| 85 (21.9) \| 10 (20.4) \| 0.66 \| \| Smoking status \|  \|  \|  \| 0.05 \| \| *Yes* \| 45 (10.3) \| 36 (9.3) \| 9 (18.4) \|  \| \| *Ex-smoker* \| 242 (55.5) \| 213 (55.0) \| 29 (59.2) \|  \| \| *No* \| 149 (34.2) \| 138 (35.7) \| 11 (22.4) \|  \| \| Baseline AF \| 73 (16.7) \| 60 (15.5) \| 13 (26.5) \| 0.05 \| \| NYHA functional class \|  \|  \|  \| 0.47 \| \| *I* \| 145 (33.3) \| 132 (34.2) \| 13 (26.5) \|  \| \| *II* \| 197 (45.3) \| 171 (44.3) \| 26 (53.1) \|  \| \| *III or IV* \| 93 (21.4) \| 83 (21.5) \| 10 (20.4) \|  \| \| **Medications** \|  \|  \|  \|  \| \| Antithrombotic therapy \| 420 (96.1) \| 371 (95.6) \| 49 (100.0) \| 0.14 \| \| Beta-blocker \| 338 (77.3) \| 299 (77.1) \| 39 (79.6) \| 0.69 \| \| ACEi/ARB \| 368 (84.2) \| 320 (82.5) \| 48 (98.0) \| 0.005 \| \| Lipid-lowering drug \| 386 (88.3) \| 340 (87.6) \| 46 (93.9) \| 0.20 \| \| **CMR volumetric measurements** \|  \|  \|  \|  \| \| LVEF (%) \| 47 (17) \| 49 (17) \| 37 (14) \| <0.001 \| \| Indexed LV mass (g/m2) \| 79 (24) \| 78 (24) \| 91 (27) \| <0.001 \| \| LVEDVi (ml/m2) \| 107 (41) \| 103 (38) \| 136 (50) \| <0.001 \| \| RVEF (%) \| 58 (13) \| 59 (12) \| 55 (15) \| 0.08 \| \| **CMR LGE characteristics** \|  \|  \|  \|  \| \| Presence of infarct pattern LGE \| 378 (86.5) \| 329 (84.8) \| 49 (100.0) \| 0.003 \| \| Predominant territory \|  \|  \|  \| 0.75 \| \| *Anterior* \| 172 (45.5) \| 152 (46.2) \| 20 (40.8) \|  \| \| *Lateral* \| 54 (14.3) \| 47 (14.3) \| 7 (14.3) \|  \| \| *Inferior* \| 152 (40.2) \| 130 (39.5) \| 22 (44.9) \|  \| \| PIZ mass (g), median (IQR) \| 8.8 (4.2-14.4) \| 7.9 (3.4-13.1) \| 15.8 (10.8-23.1) \| <0.001 \| \| Infarct core mass (g), median (IQR) \| 17.6 (6.5-30.2) \| 15.6 (5.0-27.5) \| 34.2 (23.6-44.2) \| <0.001 \| \| ACEi = angiotensin-converting enzyme inhibitor; AF = atrial fibrillation; ARB = angiotensin II receptor blocker; aSCD = aborted sudden cardiac death; BMI = body mass index; CABG = coronary artery bypass grafting; CAD = coronary artery disease; CMR = cardiovascular magnetic resonance; DBP = diastolic blood pressure; IQR = interquartile range; LGE = late gadolinium enhancement; LVEDVi = indexed left ventricular end-diastolic volume; LVEF = left ventricular ejection fraction; MI = myocardial infarction; NA = not applicable; NYHA = New York Heart Association; RVEDVi = indexed right ventricular end-systolic volume; RVEF = right ventricular ejection fraction; PIZ = peri-infarct zone; PCI = percutaneous coronary intervention; SCD = sudden cardiac death; SBP = systolic blood pressure. *Patients with evidence of severe CAD or a history of prior coronary revascularisation.  Continuous variables are reported as mean (standard deviation) or median (interquartile range). Categorical variables are reported as N (%). \| \| \| \| \| |
| --- | --- | --- | --- | --- | --- | --- | --- | --- | --- | --- | --- | --- | --- | --- | --- | --- | --- | --- | --- | --- | --- | --- | --- | --- | --- | --- | --- | --- | --- | --- | --- | --- | --- | --- | --- | --- | --- | --- | --- | --- | --- | --- | --- | --- | --- | --- | --- | --- | --- | --- | --- | --- | --- | --- | --- | --- | --- | --- | --- | --- | --- | --- | --- | --- | --- | --- | --- | --- | --- | --- | --- | --- | --- | --- | --- | --- | --- | --- | --- | --- | --- | --- | --- | --- | --- | --- | --- | --- | --- | --- | --- | --- | --- | --- | --- | --- | --- | --- | --- | --- | --- | --- | --- | --- | --- | --- | --- | --- | --- | --- | --- | --- | --- | --- | --- | --- | --- | --- | --- | --- | --- | --- | --- | --- | --- | --- | --- | --- | --- | --- | --- | --- | --- | --- | --- | --- | --- | --- | --- | --- | --- | --- | --- | --- | --- | --- | --- | --- | --- | --- | --- | --- | --- | --- | --- | --- | --- | --- | --- | --- | --- | --- | --- | --- | --- | --- | --- | --- | --- | --- | --- | --- | --- | --- | --- | --- | --- | --- | --- | --- | --- | --- | --- | --- | --- | --- | --- | --- | --- | --- | --- | --- | --- | --- | --- | --- | --- | --- | --- | --- | --- | --- | --- | --- | --- | --- | --- | --- | --- | --- | --- | --- | --- | --- | --- | --- | --- | --- | --- | --- | --- | --- | --- | --- | --- | --- | --- | --- | --- | --- | --- | --- | --- | --- | --- | --- | --- | --- | --- | --- | --- | --- | --- | --- | --- | --- | --- | --- | --- | --- |

| **Supplemental Table 2: Descriptive statistics for the scar microstructure features, stratified by the primary outcome.** | | | | |
| --- | --- | --- | --- | --- |
| **Scar feature** | **All Patients** | **No SCD/aSCD** | **SCD/aSCD** | **P-value** |
| **Interface area – total LGE interface area across all slices** | | | | |
| Core infarct interface area | 92.0 (85.8 - 98.2) | 84.5 (78.3 - 90.7) | 151.4 (132.1 - 170.7) | <0.001 |
| PIZ interface area | 139.9 (130.0 - 149.8) | 129.6 (119.7 - 139.6) | 220.8 (187.2 - 254.5) | <0.001 |
| Core infarct & PIZ combined interface area | 107.0 (99.8 - 114.2) | 99.6 (92.3 - 106.8) | 166.1 (143.7 - 188.5) | <0.001 |
| **Entropy – mean LGE entropy across all slices** | | | | |
| Core infarct entropy | 2.3 (2.2 - 2.4) | 2.2 (2.1 - 2.3) | 2.9 (2.8 - 3.1) | <0.001 |
| PIZ entropy | 1.6 (1.5 - 1.7) | 1.5 (1.5 - 1.6) | 2.0 (1.9 - 2.1) | <0.001 |
| Core infarct & PIZ combined entropy | 2.5 (2.4 - 2.6) | 2.4 (2.3 - 2.5) | 3.2 (3.0 - 3.3) | <0.001 |
| **Transmurality – mean transmurality of LGE across all slices** | | | | |
| Core infarct transmurality | 0.3 (0.3 - 0.4) | 0.3 (0.3 - 0.3) | 0.5 (0.4 - 0.5) | <0.001 |
| PIZ transmurality | 0.2 (0.2 - 0.2) | 0.2 (0.2 - 0.2) | 0.3 (0.2 - 0.3) | <0.001 |
| Core infarct & PIZ combined transmurality | 0.4 (0.4 - 0.4) | 0.4 (0.4 - 0.4) | 0.6 (0.5 - 0.6) | <0.001 |
| **Number of components – total number of LGE components across all slices** | | | | |
| Number of core infarct components | 4.0 (3.7 - 4.3) | 3.8 (3.5 - 4.1) | 5.6 (4.8 - 6.4) | <0.001 |
| Number of PIZ components | 12.8 (12.0 - 13.7) | 12.0 (11.1 - 12.9) | 19.3 (16.8 - 21.8) | <0.001 |
| Number of core infarct & PIZ combined components | 4.7 (4.4 - 5.0) | 4.5 (4.2 - 4.8) | 6.5 (5.5 - 7.6) | <0.001 |
| **Radiality – mean radiality of LGE across all slices** | | | | |
| Core infarct radiality | 0.2 (0.2 - 0.2) | 0.2 (0.2 - 0.2) | 0.3 (0.2 - 0.3) | <0.001 |
| PIZ radiality | 0.3 (0.2 - 0.3) | 0.2 (0.2 - 0.3) | 0.4 (0.3 - 0.4) | <0.001 |
| Core infarct & PIZ combined radiality | 0.2 (0.2 - 0.3) | 0.2 (0.2 - 0.2) | 0.3 (0.3 - 0.4) | <0.001 |
| Mean value (95% confidence interval) for the scar microstructure features; all patients and stratified for the primary outcome. PIZ = peri-infarct zone | | | | |

| **Supplemental Table 3: Reproducibility analysis for the scar quantification data** | | | |
| --- | --- | --- | --- |
|  | Intraobserver ICC | Interobserver ICC | ICC between serial scans* |
| Core infarct mass | 0.85 | 0.85 | 0.77 |
| PIZ mass | 0.90 | 0.77 | 0.87 |
| Reproducibility data for core infarct mass and PIZ mass quantification using intraclass correlation coefficient calculation. * calculated in patients who had 2 clinical scans without documented evidence of an incident myocardial infarction between studies.  ICC = intraclass correlation coefficient; PIZ = peri-infarct mass | | | |

| **Supplemental Table 4: Summary of univariable and multivariable Cox regression analysis for the primary endpoint.** |
| --- |
| \|  \| **Univariable analysis** \| \| **Multivariable analysis** \| \| \| \| \| --- \| --- \| --- \| --- \| --- \| --- \| --- \| \| **Model A^+^** \| \| **Model B^++^** \| \| \| **Variable** \| **HR (95% CI)** \| **P-value** \| **HR (95% CI)** \| **P-value** \| **HR (95% CI)** \| **P-value** \| \| **Demographics** \|  \|  \|  \|  \|  \|  \| \| Age (per 10 years) \| 0.83 (0.63, 1.10) \| 0.19 \| - \| - \| 0.96 (0.71, 1.29) \| 0.78 \| \| Female \| 0.24 (0.06, 0.99) \| 0.05 \| - \| - \| 0.33 (0.08, 1.39) \| 0.13 \| \| Caucasian \| 0.88 (0.45, 1.72) \| 0.71 \| - \| - \| - \| - \| \| BMI (kg/m2) \| 1.05 (1.00, 1.11) \| 0.04 \| - \| - \| - \| - \| \| Heart rate (per 10 bpm) \| 1.05 (0.86, 1.30) \| 0.62 \| - \| - \| - \| - \| \| SBP (per 10 mmHg) \| 0.88 (0.75, 1.03) \| 0.11 \| - \| - \| - \| - \| \| DBP (per 10 mmHg) \| 0.88 (0.69, 1.12) \| 0.30 \| - \| - \| - \| - \| \| Significant CAD* \| 2.32 (0.32, 16.84) \| 0.40 \| - \| - \| - \| - \| \| CAD type \|  \| 0.65 \| - \| - \| - \| - \| \| *Single vessel* \| 0.77 (0.38, 1.57) \|  \|  \|  \|  \|  \| \| *2 vessels* \| 1.08 (0.55, 2.13) \|  \|  \|  \|  \|  \| \| *3 vessels* \| Reference group \|  \|  \|  \|  \|  \| \| History of MI \| 2.08 (0.97, 4.43) \| 0.06 \| - \| - \| - \| - \| \| Prior PCI \| 0.59 (0.34, 1.05) \| 0.07 \| - \| - \| - \| - \| \| Prior CABG \| 1.62 (0.91, 2.90) \| 0.10 \| - \| - \| - \| - \| \| Hypertension \| 1.00 (0.57, 1.75) \| 1.00 \| - \| - \| - \| - \| \| Diabetes mellitus \| 1.48 (0.82, 2.67) \| 0.19 \| - \| - \| - \| - \| \| Documented hypercholesterolemia \| 1.32 (0.59, 2.94) \| 0.50 \| - \| - \| - \| - \| \| Documented family history of premature CAD \| 0.87 (0.62, 1.22) \| 0.42 \| - \| - \| - \| - \| \| Smoking status \|  \| 0.01 \| - \| - \|  \| 0.04 \| \| *Yes* \| Reference group \|  \|  \|  \| Reference group \|  \| \| *Ex-smoker* \| 1.74 (0.87, 3.50) \|  \|  \|  \| 1.30 (0.64, 2.65) \|  \| \| *No* \| 3.24 (1.34, 7.82) \|  \|  \|  \| 2.71 (1.08, 6.80) \|  \| \| Baseline AF \| 1.95 (1.04, 3.69) \| 0.04 \| - \| - \| 2.37 (1.19, 4.75) \| 0.02 \| \| NYHA functional class \|  \|  \| - \| - \| - \| - \| \| *II, III or IV* \| 1.60 (0.85, 3.02) \| 0.15 \| 1.20 (0.63, 2.30) \| 0.58 \| - \| - \| \| **Medications** \|  \|  \|  \|  \|  \|  \| \| Antithrombotic therapy \| 6.2e+14 (0.00, .) \| 1.00 \| - \| - \| - \| - \| \| Beta-blocker \| 1.24 (0.62, 2.48) \| 0.55 \| - \| - \| - \| - \| \| ACEi/ARB \| 10.06 (1.39, 72.93) \| 0.02 \| - \| - \| 4.70 (0.64, 34.57) \| 0.13 \| \| Lipid-lowering drug \| 1.94 (0.60, 6.23) \| 0.27 \| - \| - \| - \| - \| \| **CMR volumetric measurements** \|  \|  \|  \|  \|  \|  \| \| LVEF (%) \| 0.96 (0.94, 0.97) \| <0.001 \| - \| - \| 0.99 (0.97, 1.02) \| 0.63 \| \| LVEF <35% \| 3.22 (1.84, 5.65) \| <0.001 \| 1.65 (0.90, 3.03) \| 0.11 \| - \| - \| \| Indexed LV mass (g/m2) \| 1.02 (1.01, 1.03) \| <0.001 \| - \| - \| - \| - \| \| LVEDVi (ml/m2) \| 8.25 (3.92, 17.36) \| <0.001 \| - \| - \| - \| - \| \| RVEF (%) \| 0.98 (0.96, 1.00) \| 0.03 \| - \| - \| - \| - \| \| **CMR LGE characteristics** \|  \|  \|  \|  \|  \|  \| \| Presence of infarct pattern LGE \| 7.6e+14 (0.00, .) \| 1.00 \| - \| - \| - \| - \| \| Predominant territory \|  \| 0.71 \| - \| - \| - \| - \| \| *Anterior* \| Reference group \|  \|  \|  \|  \|  \| \| *Lateral* \| 1.16 (0.49, 2.75) \|  \|  \|  \|  \|  \| \| *Inferior* \| 1.29 (0.70, 2.36) \|  \|  \|  \|  \|  \| \| PIZ mass (g) \| 1.12 (1.09, 1.15) \| <0.001 \| 1.07 (1.02, 1.12) \| 0.002 \| 1.07 (1.02, 1.12) \| 0.005 \| \| Infarct core mass (g) \| 1.05 (1.04, 1.06) \| <0.001 \| 1.03 (1.01, 1.05) \| 0.01 \| 1.02 (1.00, 1.05) \| 0.03 \| \| ACEi = angiotensin-converting enzyme inhibitor; AF = atrial fibrillation; ARB = angiotensin II receptor blocker; BMI = body mass index; CABG = coronary artery bypass grafting; CAD = coronary artery disease; CI = confidence interval; CMR = cardiovascular magnetic resonance; DBP = diastolic blood pressure; HR = hazard ratio; LGE = late gadolinium enhancement; LVEDVi = indexed left ventricular end-diastolic volume; LVEF = left ventricular ejection fraction; MI = myocardial infarction; NYHA = New York Heart Association; RVEF = right ventricular ejection fraction; PIZ = peri-infarct zone; PCI = percutaneous coronary intervention; SBP = systolic blood pressure. *Patients with evidence of severe CAD or a history of prior coronary revascularisation. ^+^Model A: To align with current clinical guidelines for ICD implantation, a multivariable model using binary cut-offs of LVEF <35% and NYHA class >1 was generated with subsequent addition of the LGE quantification data. ^++^Model B: To select this model, a forward stepwise procedure was applied with p<0.10 as the criterion for inclusion, forcing in recognised predictors of the outcome (age, sex and LVEF) with simultaneous addition of the LGE quantification data. A limited number of robust variables were chosen to prevent creating an overly complex model. Not all variables that were associated with the primary endpoint on univariable analysis were used in the multivariable model (e.g., indexed LV mass and RVEF). \| \| \| \| \| \| \| |

Supplemental Table 5: Multivariable analysis of the scar microstructure features, adjusted for LVEF <35% and NYHA >1.

| **Scar feature** | **Scar feature HR and 95% CI** | **Scar feature P-value** |
| --- | --- | --- |
| **Interface area – total LGE interface area across all slices** | | |
| Core infarct interface area | 2.0 (1.52 - 2.62) | <0.001 |
| PIZ interface area | 1.64 (1.29 - 2.09) | <0.001 |
| Core infarct & PIZ combined interface area | 1.59 (1.26 - 2.0) | <0.001 |
| **Entropy – mean LGE entropy across all slices** | | |
| Core infarct entropy | 2.4 (1.53 - 3.77) | <0.001 |
| PIZ entropy | 2.07 (1.38 - 3.09) | <0.001 |
| Core infarct & PIZ combined entropy | 2.29 (1.46 - 3.58) | <0.001 |
| **Transmurality – mean transmurality of LGE across all slices** | | |
| Core infarct transmurality | 1.89 (1.37 - 2.6) | <0.001 |
| PIZ transmurality | 1.69 (1.23 - 2.33) | 0.001 |
| Core infarct & PIZ combined transmurality | 2.23 (1.54 - 3.22) | <0.001 |
| **Number of components – total number of LGE components across all slices** | | |
| Number of core infarct components | 1.39 (1.08 - 1.8) | 0.012 |
| Number of PIZ components | 1.61 (1.27 - 2.03) | <0.001 |
| Number of core infarct & PIZ combined components | 1.46 (1.14 - 1.86) | 0.002 |
| **Radiality – mean radiality of LGE across all slices** | | |
| Core infarct radiality | 1.55 (1.18 - 2.03) | 0.001 |
| PIZ radiality | 1.65 (1.24 - 2.2) | 0.001 |
| Core infarct & PIZ combined radiality | 1.58 (1.19 - 2.09) | 0.002 |
| Multivariable Cox regression models of the primary endpoint for each scar microstructure feature, adjusted for LVEF <35% and NYHA >1. CI = confidence interval; HR = Hazard ratio; LGE = late gadolinium enhancement; PIZ = peri-infarct zone | | |

Supplemental Table 6: Multivariable analysis of the scar microstructure features, adjusted for LVEF <35%, NYHA >1, core infarct mass and PIZ mass

| **Scar feature** | **Scar feature HR and 95% CI** | **Scar feature P-value** |
| --- | --- | --- |
| **Interface area – total LGE interface area across all slices** | | |
| Core infarct interface area | 0.92 (0.63 - 1.34) | 0.648 |
| PIZ interface area | 0.9 (0.66 - 1.25) | 0.544 |
| Core infarct & PIZ combined interface area | 0.8 (0.56 - 1.15) | 0.232 |
| **Entropy – mean LGE entropy across all slices** | | |
| Core infarct entropy | 1.29 (0.74 - 2.26) | 0.369 |
| PIZ entropy | 1.05 (0.64 - 1.73) | 0.833 |
| Core infarct & PIZ combined entropy | 1.19 (0.67 - 2.09) | 0.555 |
| **Transmurality – mean transmurality of LGE across all slices** | | |
| Core infarct transmurality | 1.42 (0.9 - 2.24) | 0.135 |
| PIZ transmurality | 0.97 (0.63 - 1.5) | 0.892 |
| Core infarct & PIZ combined transmurality | 1.43 (0.84 - 2.42) | 0.185 |
| **Number of components – total number of LGE components across all slices** | | |
| Number of core infarct components | 0.76 (0.53 - 1.09) | 0.139 |
| Number of PIZ components | 0.9 (0.64 - 1.29) | 0.577 |
| Number of core infarct & PIZ combined components | 0.88 (0.6 - 1.29) | 0.517 |
| **Radiality – mean radiality of LGE across all slices** | | |
| Core infarct radiality | 0.73 (0.47 - 1.13) | 0.154 |
| PIZ radiality | 0.75 (0.48 - 1.18) | 0.218 |
| Core infarct & PIZ combined radiality | 0.65 (0.41 - 1.03) | 0.069 |
| Multivariable Cox regression models of the primary endpoint for each scar microstructure feature, adjusted for LVEF <35%, NYHA >1, core infarct mass and PIZ mass. CI = confidence interval; HR = Hazard ratio; LGE = late gadolinium enhancement; PIZ = peri-infarct zone | | |

Supplemental Table 7: Multivariable analysis of the scar microstructure features in patients with LVEF ≥35%, adjusted for age, sex and LVEF.

| **Scar feature** | **Scar feature HR and 95% CI** | **Scar feature P-value** |
| --- | --- | --- |
| **Interface area – total LGE interface area across all slices** | | |
| Core infarct interface area* | 1.52 (1.02 - 2.27) | 0.039 |
| PIZ interface area | 1.51 (1.0 - 2.28) | 0.052 |
| Core infarct & PIZ combined interface area | 1.27 (0.87 - 1.85) | 0.213 |
| **Entropy – mean LGE entropy across all slices** | | |
| Core infarct entropy* | 2.49 (1.16 - 5.33) | 0.019 |
| PIZ entropy* | 2.04 (1.07 - 3.88) | 0.029 |
| Core infarct & PIZ combined entropy* | 2.84 (1.31 - 6.17) | 0.008 |
| **Transmurality – mean transmurality of LGE across all slices** | | |
| Core infarct transmurality | 1.58 (0.9 - 2.79) | 0.113 |
| PIZ transmurality* | 2.16 (1.17 - 3.98) | 0.014 |
| Core infarct & PIZ combined transmurality* | 2.89 (1.41 - 5.94) | 0.004 |
| **Number of components – total number of LGE components across all slices** | | |
| Number of core infarct components | 1.27 (0.85 - 1.89) | 0.237 |
| Number of PIZ components | 1.18 (0.78 - 1.8) | 0.432 |
| Number of core infarct & PIZ combined components | 1.14 (0.73 - 1.79) | 0.564 |
| **Radiality – mean radiality of LGE across all slices** | | |
| Core infarct radiality | 1.22 (0.77 - 1.91) | 0.396 |
| PIZ radiality | 1.19 (0.73 - 1.93) | 0.491 |
| Core infarct & PIZ combined radiality | 1.11 (0.69 - 1.77) | 0.676 |
| Multivariable Cox regression models of the primary endpoint for each scar microstructure feature in patients with LVEF ≥35%. Each model adjusted for age, sex and LVEF. *Scar microstructure features reaching statistical significance. CI = confidence interval; HR = Hazard ratio; LGE = late gadolinium enhancement; PIZ = peri-infarct zone | | |

| Supplemental Table 8. Sensitivity, specificity and predictive values | | | | |
| --- | --- | --- | --- | --- |
|  | Sensitivity | Specificity | PPV | NPV |
| **Traditional metric** |  |  |  |  |
| LVEF <35% | 51% | 24.2% | 7.8% | 79.7% |
| **PIZ optimal threshold 11.6g** |  |  |  |  |
| Total population | 73.5% | 69.3% | 23.2% | 95.4% |
| LVEF<35% | 91.7% | 51.1% | 32.3% | 96.0% |
| LVEF≥35% | 56.0% | 75.2% | 16.1% | 95.3% |
| LVEF = left ventricular ejection fraction; NPV = negative predictive value; PIZ zone = peri-infarct zone; PPV = positive predictive value. | | | | |

| **Supplemental Table 9. Scan indication** | |
| --- | --- |
| Scan indication | Patients |
| Assessment of cardiac function and scar burden, n | 364 (83%) |
| Diagnostic uncertainty, n | 42 (10%) |
| Prior to device insertion, n | 18 (4%) |
| Other (e.g., valve assessment), n | 13 (3%) |

|  |
| --- |
| Supplemental Figure 1: Study cohort.  Flow diagram describing the curation of the study cohort. ACS = acute coronary syndrome; CAD = coronary artery disease; CMR = cardiovascular magnetic resonance; ICD = implantable cardioverter defibrillator; LGE = late gadolinium enhancement; UK = United Kingdom. |

| \|  \|  \| **LVEF & NYHA + PIZ mass** \| \| \| \|  \|  \| \| --- \| --- \| --- \| --- \| --- \| --- \| --- \| --- \| \| **10-year risk** \| \| **0-10%** \| **10-20%** \| **20%+** \| **Total** \| **Higher** \| **Lower** \| \| **LVEF & NYHA only** \| \|  \|  \|  \|  \|  \|  \| \| **SCD/aborted SCD** \| \|  \|  \|  \|  \|  \|  \| \|  \| **0-10%** \| 0 \| 0 \| 0 \| 0 \| 0 \| NA \| \|  \| **10-20%** \| 5 \| 12 \| 8 \| 25 \| 8 \| 5 \| \|  \| **20%+** \| 0 \| 1 \| 23 \| 24 \| NA \| 1 \| \|  \| **Total** \| 5 \| 13 \| 31 \| 49 \| 8 \| 6 \| \| **No SCD/aborted SCD** \| \|  \|  \|  \|  \|  \|  \| \|  \| **0-10%** \| 0 \| 0 \| 0 \| 0 \| 0 \| NA \| \|  \| **10-20%** \| 115 \| 94 \| 45 \| 294 \| 45 \| 115 \| \|  \| **20%+** \| 15 \| 22 \| 57 \| 94 \| NA \| 37 \| \|  \| **Total** \| 170 \| 116 \| 102 \| 388 \| 45 \| 152 \| \|  \|  \|  \|  \|  \|  \|  \|  \| \|  \|  \| **NRI** \| **95% CI** \|  \|  \|  \|  \| \|  \| **Categorical NRI** \|  \|  \|  \|  \|  \|  \| \|  \| Event NRI \| 0.04 \| -1 to 1 \|  \|  \|  \|  \| \|  \| Non-event NRI \| 0.28 \| -0.05 to 0.61 \|  \|  \|  \|  \| \|  \| Overall NRI \| 0.32 \| -2 to 2 \|  \|  \|  \|  \| \|  \| **Continuous NRI** \|  \|  \|  \|  \|  \|  \| \|  \| Event NRI \| 0.18 \| -0.07 to 0.43 \|  \|  \|  \|  \| \|  \| Non-event NRI \| 0.41 \| 0.28 to 0.54 \|  \|  \|  \|  \| \|  \| Overall NRI \| 0.59 \| 0.31 to 0.87 \|  \|  \|  \|  \| |
| --- | --- | --- | --- | --- | --- | --- | --- | --- | --- | --- | --- | --- | --- | --- | --- | --- | --- | --- | --- | --- | --- | --- | --- | --- | --- | --- | --- | --- | --- | --- | --- | --- | --- | --- | --- | --- | --- | --- | --- | --- | --- | --- | --- | --- | --- | --- | --- | --- | --- | --- | --- | --- | --- | --- | --- | --- | --- | --- | --- | --- | --- | --- | --- | --- | --- | --- | --- | --- | --- | --- | --- | --- | --- | --- | --- | --- | --- | --- | --- | --- | --- | --- | --- | --- | --- | --- | --- | --- | --- | --- | --- | --- | --- | --- | --- | --- | --- | --- | --- | --- | --- | --- | --- | --- | --- | --- | --- | --- | --- | --- | --- | --- | --- | --- | --- | --- | --- | --- | --- | --- | --- | --- | --- | --- | --- | --- | --- | --- | --- | --- | --- | --- | --- | --- | --- | --- | --- | --- | --- | --- | --- | --- | --- | --- | --- | --- | --- | --- | --- | --- | --- | --- | --- | --- | --- | --- | --- | --- | --- | --- | --- | --- | --- | --- | --- | --- | --- | --- | --- | --- | --- | --- | --- | --- | --- | --- | --- | --- | --- | --- | --- | --- | --- | --- |
| \|  \|  \| **LVEF & NYHA + core mass + PIZ mass** \| \| \| \|  \|  \| \| --- \| --- \| --- \| --- \| --- \| --- \| --- \| --- \| \| **10-year risk** \| \| **0-10%** \| **10-20%** \| **20%+** \| **Total** \| **Higher** \| **Lower** \| \| **LVEF & NYHA + core mass** \| \|  \|  \|  \|  \|  \|  \| \| **SCD/aborted SCD** \| \|  \|  \|  \|  \|  \|  \| \|  \| **0-10%** \| 5 \| 0 \| 0 \| 5 \| 0 \| NA \| \|  \| **10-20%** \| 1 \| 7 \| 0 \| 8 \| 0 \| 1 \| \|  \| **20%+** \| 0 \| 3 \| 33 \| 36 \| NA \| 3 \| \|  \| **Total** \| 6 \| 10 \| 33 \| 49 \| 0 \| 4 \| \| **No SCD/aborted SCD** \| \|  \|  \|  \|  \|  \|  \| \|  \| **0-10%** \| 142 \| 7 \| 0 \| 149 \| 7 \| NA \| \|  \| **10-20%** \| 34 \| 79 \| 14 \| 127 \| 14 \| 34 \| \|  \| **20%+** \| 0 \| 21 \| 91 \| 112 \| NA \| 21 \| \|  \| **Total** \| 176 \| 107 \| 195 \| 388 \| 21 \| 55 \| \|  \|  \|  \|  \|  \|  \|  \|  \| \|  \|  \| **NRI** \| **95% CI** \|  \|  \|  \|  \| \|  \| **Categorical NRI** \|  \|  \|  \|  \|  \|  \| \|  \| Event NRI \| -0.08 \| -0.73 to 0.57 \|  \|  \|  \|  \| \|  \| Non-event NRI \| 0.09 \| -0.02 to 0.20 \|  \|  \|  \|  \| \|  \| Overall NRI \| 0.01 \| -0.52 to 0.56 \|  \|  \|  \|  \| \|  \| **Continuous NRI** \|  \|  \|  \|  \|  \|  \| \|  \| Event NRI \| -0.02 \| -0.32 to 0.28 \|  \|  \|  \|  \| \|  \| Non-event NRI \| 0.46 \| 0.29 to 0.63 \|  \|  \|  \|  \| \|  \| Overall NRI \| 0.44 \| 0.14 to 0.74 \|  \|  \|  \|  \| |
| Supplemental Figure 2: Risk reclassification for the primary endpoint.  Top row: Reclassification of patient risk for the primary outcome using net reclassification indices. Predicted overall risk of SCD/aborted SCD was first determined on the basis of a model using LVEF <35% and NYHA >1. The relative improvement in patient reclassification associated with PIZ mass was then assessed. Reclassification was examined using thresholds of 0-10%, 10-20% and 20%+ to stratify level of risk (categorical NRI), in addition to calculating a category-free net reclassification index (continuous NRI).  Bottom row: Reclassification of patient risk for the primary outcome using net reclassification indices. Predicted overall risk of SCD/aborted SCD was determined on the basis of a model using LVEF <35%, NYHA >1 and core infarct mass. The relative improvement in patient reclassification associated with PIZ mass was then assessed. Reclassification was examined using thresholds of 0-10%, 10-20% and 20%+ to stratify level of risk (categorical NRI), in addition to calculating a category-free net reclassification index (continuous NRI).  CI = confidence interval; LVEF = left ventricular ejection fraction; NYHA = New York Heart Association; NRI = net reclassification index; PIZ = peri-infarct zone; SCD = sudden cardiac death. |

| \| **Variable** \| **sHR (95% CI)** \| **P-value** \| \| --- \| --- \| --- \| \| **LVEF<35%** \| 2.66 (1.51, 4.69) \| 0.001 \| \| **NYHA >1** \| 1.20 (0.64, 2.27) \| 0.57 \| | \| **Variable** \| **sHR (95% CI)** \| **P-value** \| \| --- \| --- \| --- \| \| **PIZ mass (g)** \| 1.07 (1.03, 1.12) \| 0.001 \| \| **Infarct core mass (g)** \| 1.03 (1.01, 1.04) \| 0.003 \| \| **LVEF<35%** \| 1.42 (0.80, 2.53) \| 0.23 \| \| **NYHA >1** \| 1.09 (0.56, 2.11) \| 0.80 \| |
| --- | --- | --- | --- | --- | --- | --- | --- | --- | --- | --- | --- | --- | --- | --- | --- | --- | --- | --- | --- | --- | --- | --- | --- | --- | --- |
| Supplemental Figure 3: Competing risk analysis.  Competing risk survival analysis using Fine-Gray subdistribution Hazard modelling; SCD/aborted SCD versus non-sudden death. CI = confidence interval; sHR = subdistribution hazard ratio; LVEF = left ventricular ejection fraction; NYHA = New York Heart Association; PIZ = peri-infarct zone. | |

| 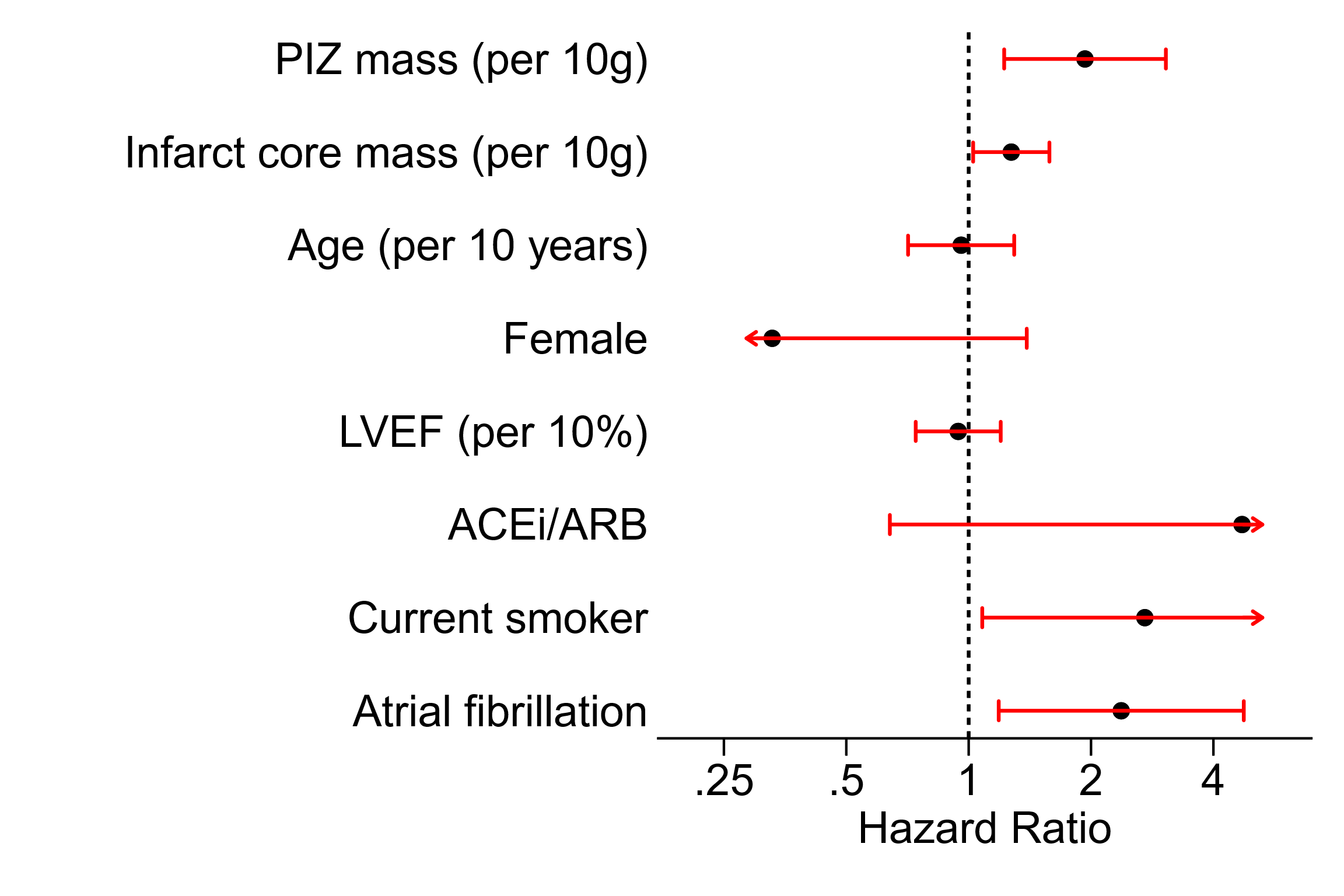 | |
| --- | --- |
| \| **Variable** \| **HR (95% CI)** \| **P-value** \| \| --- \| --- \| --- \| \| **Age (per 10 years)** \| 0.76 (0.57, 1.02) \| 0.07 \| \| **Female** \| 0.31 (0.07, 1.28) \| 0.11 \| \| **LVEF (%)** \| 0.96 (0.95, 0.98) \| <0.001 \| \| **ACE-i/ARB** \| 6.32 (0.87, 46.15) \| 0.07 \| \| **Smoking status** \|  \| 0.04 \| \| Non-smoker \| Reference group \|  \| \| Ex-smoker \| 1.57 (0.77, 3.17) \|  \| \| Current smoker \| 2.61 (1.05, 6.44) \|  \| \| **Atrial fibrillation** \| 1.98 (1.01, 3.90) \| 0.05 \| \| **Harrell's C-statistic = 0.76** \| \| \| | \| **Variable** \| **HR (95% CI)** \| **P-value** \| \| --- \| --- \| --- \| \| **Infarct core mass (g)** \| 1.04 (1.02, 1.06) \| <0.001 \| \| **Age (per 10 years)** \| 0.89 (0.66, 1.21) \| 0.46 \| \| **Female** \| 0.37 (0.09, 1.53) \| 0.17 \| \| **LVEF (%)** \| 0.99 (0.96, 1.01) \| 0.23 \| \| **ACE-i/ARB** \| 5.24 (0.71, 38.47) \| 0.10 \| \| **Smoking status** \|  \| 0.06 \| \| Non-smoker \| Reference group \|  \| \| Ex-smoker \| 1.54 (0.77, 3.12) \|  \| \| Current smoker \| 2.36 (0.94, 5.92) \|  \| \| **Atrial fibrillation** \| 2.51 (1.25, 5.03) \| 0.009 \| \| **Harrell's C-statistic = 0.81** \| \| \| |
| \| **Variable** \| **HR (95% CI)** \| **P-value** \| \| --- \| --- \| --- \| \| **PIZ mass (g)** \| 1.10 (1.06, 1.14) \| <0.001 \| \| **Age (per 10 years)** \| 0.94 (0.70, 1.27) \| 0.69 \| \| **Female** \| 0.25 (0.06, 1.08) \| 0.06 \| \| **LVEF (%)** \| 0.99 (0.97, 1.01) \| 0.33 \| \| **ACE-i/ARB** \| 5.05 (0.69, 37.05) \| 0.11 \| \| **Smoking status** \|  \| 0.06 \| \| Non-smoker \| Reference group \|  \| \| Ex-smoker \| 1.20 (0.59, 2.46) \|  \| \| Current smoker \| 2.75 (1.10, 6.88) \|  \| \| **Atrial fibrillation** \| 2.04 (1.04, 3.99) \| 0.04 \| \| **Harrell's C-statistic = 0.81** \| \| \| | \| **Variable** \| **HR (95% CI)** \| **P-value** \| \| --- \| --- \| --- \| \| **PIZ mass (g)*** \| 1.07 (1.02, 1.12) \| 0.005 \| \| **Infarct core mass (g)*** \| 1.02 (1.00, 1.05) \| 0.03 \| \| **Age (per 10 years)** \| 0.96 (0.71, 1.29) \| 0.78 \| \| **Female** \| 0.33 (0.08, 1.39) \| 0.13 \| \| **LVEF (%)** \| 0.99 (0.97, 1.02) \| 0.63 \| \| **ACE-i/ARB** \| 4.70 (0.64, 34.57) \| 0.13 \| \| **Smoking status** \|  \| 0.04 \| \| Non-smoker \| Reference group \|  \| \| Ex-smoker \| 1.30 (0.64, 2.65) \|  \| \| Current smoker \| 2.71 (1.08, 6.80) \|  \| \| **Atrial fibrillation** \| 2.37 (1.19, 4.75) \| 0.02 \| \| **Harrell's C-statistic = 0.82** \| \| \| |
| Supplemental Figure 4: Multivariable Cox regression analysis (Model B) for the primary endpoint with subsequent addition of the LGE quantification data.  In Model B, a forward stepwise procedure was applied using a subset of variables in Table 2 (with p<0.10 as the criterion for inclusion), forcing in recognised predictors of the outcome (age, sex and LVEF) and with the subsequent addition of LGE quantification data. Top row: Forest plot of the final multivariable Cox regression mode showing the simultaneous addition of the LGE quantification data. Middle and bottom rows: Multivariable Cox models for the primary endpoint with progressive addition of the LGE quantification data. *Results per 10g: PIZ mass HR 1.93, 95% CI 1.22-3.06, P=0.005 and core infarct mass HR 1.27, 95% CI 1.03-1.58, P=0.03. ACEi = angiotensin-converting enzyme inhibitor; ARB = angiotensin II receptor blocker; CI = confidence interval; HR = hazard ratio; LVEF = left ventricular ejection fraction; PIZ = peri-infarct zone. | |

| 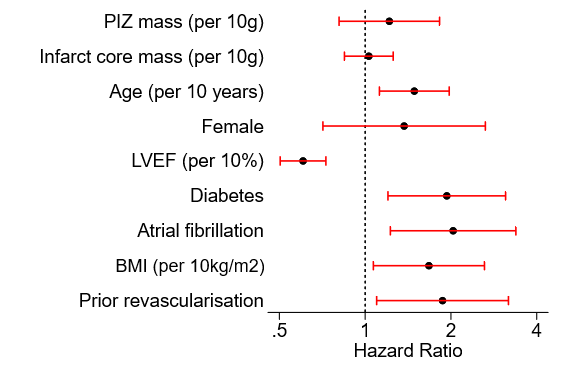 | |
| --- | --- |
| \| **Variable** \| **HR (95% CI)** \| **P-value** \| \| --- \| --- \| --- \| \| **Age (per 10 years)** \| 1.42 (1.07, 1.87) \| 0.01 \| \| **Female** \| 1.35 (0.70, 2.61) \| 0.37 \| \| **LVEF (%)** \| 0.95 (0.93, 0.96) \| <0.001 \| \| **Diabetes** \| 1.93 (1.20, 3.11) \| 0.007 \| \| **Atrial fibrillation** \| 1.98 (1.20, 3.25) \| 0.007 \| \| **BMI (kg/m^2^)** \| 1.06 (1.01, 1.11) \| 0.01 \| \| **Prior revascularisation** \| 1.94 (1.14, 3.30) \| 0.02 \| \| **Harrell's C-statistic = 0.79** \| \| \| | \| **Variable** \| **HR (95% CI)** \| **P-value** \| \| --- \| --- \| --- \| \| **PIZ mass (g)*** \| 1.02 (0.98, 1.06) \| 0.35 \| \| **Infarct core mass (g)*** \| 1.00 (0.98, 1.02) \| 0.77 \| \| **Age (per 10 years)** \| 1.49 (1.12, 1.97) \| 0.006 \| \| **Female** \| 1.37 (0.71, 2.64) \| 0.35 \| \| **LVEF (%)** \| 0.95 (0.93, 0.97) \| <0.001 \| \| **Diabetes** \| 1.93 (1.20, 3.11) \| 0.007 \| \| **Atrial fibrillation** \| 2.04 (1.23, 3.38) \| 0.006 \| \| **BMI (kg/m^2^)** \| 1.05 (1.01, 1.10) \| 0.02 \| \| **Prior revascularisation** \| 1.87 (1.10, 3.18) \| 0.02 \| \| **Harrell's C-statistic = 0.79** \| \| \| |
| Supplemental Figure 5: Multivariable analysis of major heart failure events with subsequent addition of the LGE quantification data.  Top row: Multivariable Cox regression models for major heart failure events applying a forward stepwise procedure with subsequent addition of the LGE metrics. A p<0.10 was used as the criterion for variable inclusion, forcing in recognised predictors of the outcome (age, sex and LVEF). Bottom row: Forest plot of the final multivariable model presented using LGE results per 10grams. *****Results per 10g: PIZ mass HR 1.22, 95% CI 0.81-1.82, P=0.35 and core infarct mass HR 1.03, 95% CI 0.85-1.25, P=0.77. BMI = body mass index; CI = confidence interval; HR = hazard ratio; LVEF = left ventricular ejection fraction; PIZ = peri-infarct zone. | |

| 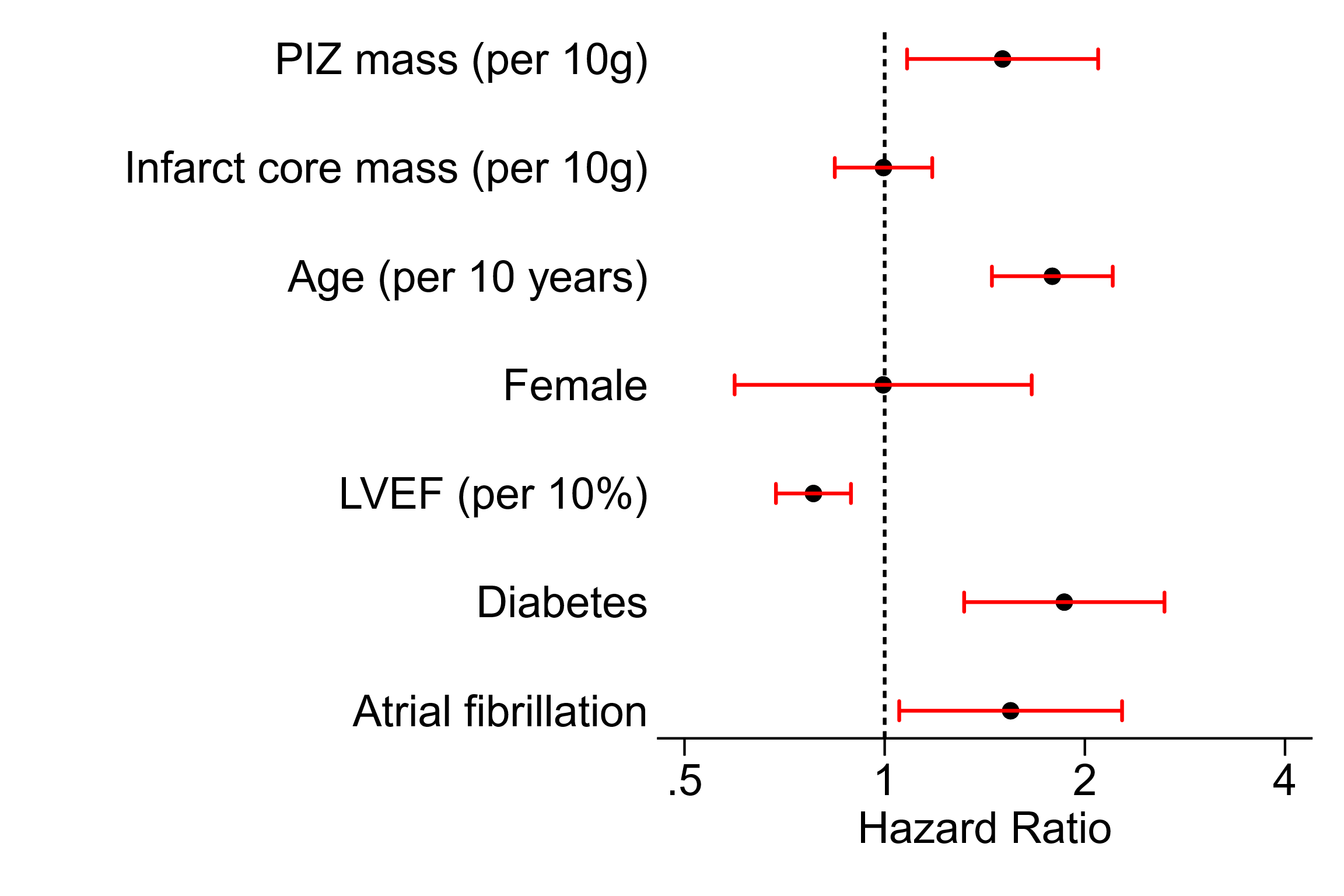 | |
| --- | --- |
| \| **Variable** \| **HR (95% CI)** \| **P-value** \| \| --- \| --- \| --- \| \| **Age (per 10 years)** \| 1.64 (1.34, 2.00) \| <0.001 \| \| **Female** \| 0.93 (0.56, 1.56) \| 0.79 \| \| **LVEF (%)** \| 0.97 (0.96, 0.98) \| <0.001 \| \| **Diabetes** \| 1.87 (1.32, 2.64) \| <0.001 \| \| **Atrial fibrillation** \| 1.53 (1.04, 2.24) \| 0.03 \| \| **Harrell's C-statistic = 0.74** \| \| \| | \| **Variable** \| **HR (95% CI)** \| **P-value** \| \| --- \| --- \| --- \| \| **PIZ mass (g)*** \| 1.04 (1.01, 1.08) \| 0.02 \| \| **Infarct core mass (g)*** \| 1.00 (0.98, 1.02) \| 0.96 \| \| **Age (per 10 years)** \| 1.79 (1.45, 2.20) \| <0.001 \| \| **Female** \| 0.99 (0.59, 1.66) \| 0.98 \| \| **LVEF (%)** \| 0.98 (0.96, 0.99) \| <0.001 \| \| **Diabetes** \| 1.86 (1.32, 2.64) \| <0.001 \| \| **Atrial fibrillation** \| 1.55 (1.05, 2.28) \| 0.03 \| \| **Harrell's C-statistic = 0.75** \| \| \| |
| Supplemental Figure 6: Multivariable analysis of all-cause mortality with subsequent addition of the LGE quantification data.  Top row: Multivariable Cox regression models for all-cause mortality applying a forward stepwise procedure with subsequent addition of the LGE metrics. A p<0.10 was used as the criterion for variable inclusion, forcing in recognised predictors of the outcome (age, sex and LVEF). Bottom row: Forest plot of the final multivariable model presented using LGE results per 10grams. *Results per 10g: PIZ mass HR 1.50, 95% CI 1.08-2.09, P=0.02 and core infarct mass HR 1.00, 95% CI 0.84-1.18, P=0.96). CI = confidence interval; HR = hazard ratio; LVEF = left ventricular ejection fraction; PIZ = peri-infarct zone. | |

| **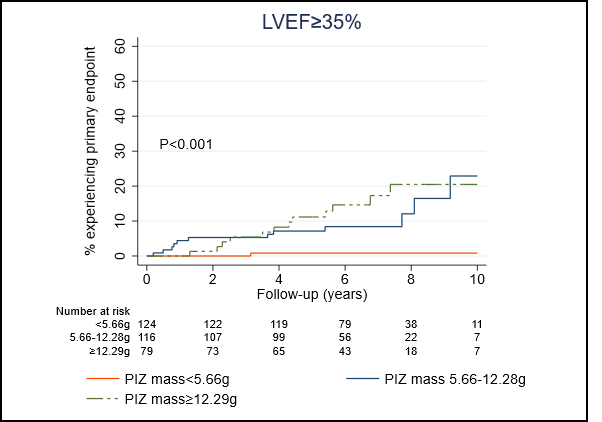** | **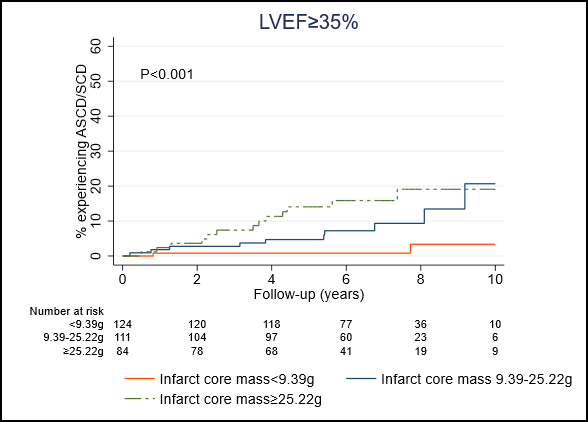** |
| --- | --- |
| \| **Variable** \| **HR (95% CI)** \| **P-value** \| \| --- \| --- \| --- \| \| **Age (per 10 years)** \| 0.89 (0.59, 1.33) \| 0.57 \| \| **Female** \| 0.25 (0.03, 1.83) \| 0.17 \| \| **LVEF (%)** \| 0.95 (0.91, 0.98) \| 0.003 \| \| **Harrell's C-statistic = 0.69** \| \| \| | \| **Variable** \| **HR (95% CI)** \| **P-value** \| \| --- \| --- \| --- \| \| **PIZ mass (g)** \| 1.02 (0.94, 1.11) \| 0.58 \| \| **Infarct core mass (g)** \| 1.03 (0.99, 1.07) \| 0.13 \| \| **Age (per 10 years)** \| 1.07 (0.70, 1.65) \| 0.76 \| \| **Female** \| 0.26 (0.03, 1.90) \| 0.18 \| \| **LVEF (%)** \| 0.98 (0.93, 1.03) \| 0.38 \| \| **Harrell's C-statistic = 0.76** \| \| \| |
| Supplemental Figure 7: Kaplan-Meier plots and multivariable models for the primary endpoint in patients with LVEF ≥35%.  Top row: Kaplan-Meier plots of the primary endpoint by tertiles of PIZ mass and core infarct mass in patients with LVEF≥ 35%. Bottom row: Multivariable Cox regression models for the primary endpoint in patients with LVEF≥ 35%, with subsequent addition of PIZ mass and core infarct mass. ASCD = aborted sudden cardiac death; CI = confidence interval; HR = hazard ratio; LVEF = left ventricular ejection fraction; PIZ = peri-infarct zone. | |

Supplemental References

1. Assomull RG., Prasad SK., Lyne J., et al. Cardiovascular magnetic resonance, fibrosis, and prognosis in dilated cardiomyopathy. J Am Coll Cardiol 2006;48(10):1977–85. Doi: 10.1016/j.jacc.2006.07.049.

2. Balaban G., Halliday BP., Bai W., et al. Scar shape analysis and simulated electrical instabilities in a non-ischemic dilated cardiomyopathy patient cohort. PLOS Computational Biology 2019;15(10):e1007421. Doi: 10.1371/journal.pcbi.1007421.

3. Balaban G., Halliday BP., Porter B., et al. Late-Gadolinium Enhancement Interface Area and Electrophysiological Simulations Predict Arrhythmic Events in Patients With Nonischemic Dilated Cardiomyopathy. JACC: Clinical Electrophysiology 2021;7(2):238–49. Doi: 10.1016/j.jacep.2020.08.036.

# 4. Hicks KA., Tcheng JE., Bozkurt B., et al. 2014 ACC/AHA Key Data Elements and Definitions for Cardiovascular Endpoint Events in Clinical Trials: A Report of the American College of Cardiology/American Heart Association Task Force on Clinical Data Standards (Writing Committee to Develop Cardiovascular Endpoints Data Standards). J Am Coll Cardiol 2015;66(4):403–69. Doi: 10.1016/j.jacc.2014.12.018.

5. Buxton AE., Calkins H., Callans DJ., et al. ACC/AHA/HRS 2006 key data elements and definitions for electrophysiological studies and procedures: a report of the American College of Cardiology/American Heart Association Task Force on Clinical Data Standards (ACC/AHA/HRS Writing Committee to Develop D. J Am Coll Cardiol 2006;48(11):2360–96. Doi: 10.1016/j.jacc.2006.09.020.

6. Zegard A., Okafor O., de Bono J., et al. Myocardial Fibrosis as a Predictor of Sudden Death in Patients With Coronary Artery Disease. J Am Coll Cardiol 2021;77(1):29–41. Doi: 10.1016/j.jacc.2020.10.046.
